# Chemical and Structural Transformations of *M*–Al–CO_3_ Layered Double Hydroxides (*M* = Mg, Zn, or Co, *M*/Al = 2) at Elevated Temperatures: Quantitative Descriptions and Effect of Divalent Cations

**DOI:** 10.1021/acs.inorgchem.4c01186

**Published:** 2024-08-12

**Authors:** Kaito Matsuda, Ayaka Okuda, Nana Iio, Naoki Tarutani, Kiyofumi Katagiri, Kei Inumaru

**Affiliations:** Graduate School of Advanced Science and Engineering, Hiroshima University, 1-4-1, Kagamiyama, Higashihiroshima, Hiroshima 739-8527, Japan

## Abstract

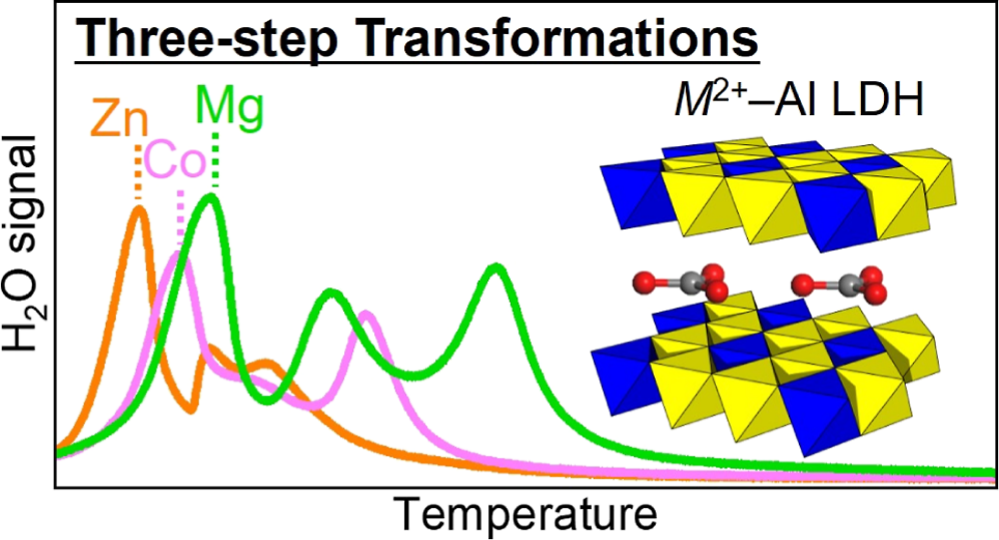

Layered double hydroxides (LDHs) exhibit diverse chemical compositions and are being designed for promising applications such as CO_2_ adsorbents. Although many researchers have analyzed CO_2_ gas evolution and structural transformation behavior at elevated temperatures, there are still inconsistencies in results on the effect of different metal ions in LDHs. In this study, on the basis of atomic/molecular-level findings from our previous study on multistep structural/chemical transformation of Mg–Al LDHs, we analyzed the quantitative gas evolution behavior and structural transformations of *M*–Al–CO_3_ LDHs with different divalent metal ions (*M* = Mg, Zn, or Co, *M*/Al = 2) at elevated temperatures. Our quantitative analysis revealed that all three LDH samples undergo the three-step chemical transformations: release of interlayer water, partial dehydroxylation of the hydroxyl layers, and complete dehydroxylation of layers and decomposition of interlayer CO_3_^2–^. However, the temperature range for each step differs, as do the structural transformations for each sample: the layered structure collapses in the first step for Zn–Al LDH and Co–Al LDH, and the third step for Mg–Al LDH. Our results provide for quantitative and concrete understanding of the effect of divalent metal ions in LDHs on thermal decomposition.

## Abstract 2

Based on our previous concrete interpretation of the multistep transformation behavior for Mg–Al LDHs, quantitative analysis of gas evolutions and in situ XRD and FT-IR measurements revealed that *M*–Al–CO_3_ LDHs with different divalent metal ions (*M* = Mg, Zn, or Co, *M*/Al = 2) undergo the same three-step chemical transformations, although the temperature range for each step differs. This study provides rational insights into the effect of divalent metal ions in LDHs on their thermal decomposition behavior.

## Introduction

Layered double hydroxides (LDHs) are compound structures comprising positively charged metal hydroxide layers, interlayer water, and anions. The general chemical formula for LDHs is *M*_1–*x*_^2+^*M*_*x*_^3+^ (OH)_2_*A*_*x/n*_^*n*–^·*m*H_2_O. The positive charge density of the metal hydroxide layer is determined by the *M*^2+^/*M*^3+^ ratio, and *n*-valent A^*n*–^ anions are incorporated in the interlayer spaces to neutralize the positive charge. LDHs can be formed by various metal ions, *M*^2+^ representing divalent cations such as Mg^2+^, Co^2+^, Ni^2+^ or Zn^2+^, and *M*^3+^ denoting Al^3+^, Ga^3+^, Fe^3+^, or other trivalent cations, including transition metal ions. LDHs can accommodate various species as interlayer anions, such as CO_3_^2–^, NO_3_^–^, and Cl^–^.^1^

Because of their diverse chemical compositions, LDHs have been extensively studied for a wide range of applications. Layered nickel hydroxides and Fe-containing LDHs show promise as electrode catalysts for water oxidation.^2,3^ LDHs also find applications in solid base catalysis for organic chemical reactions,^4−6^ photocatalysis for CO_2_ reduction,^7,8^ and ion storage materials,^9^ contributing to green chemistry and sustainable energy research.^10^ Recent developments have enabled the synthesis of LDH nanomaterials, such as nanocrystals^11−14^ and nanosheets.^15^

LDHs are recognized as promising CO_2_ adsorbents.^1,16−19^ They adsorb ambient CO_2_ to form CO_3_^2–^ interlayer anions. These CO_3_^2–^ anions form stable hydrogen-bonding networks with water molecules in the interlayer spaces^20^ and can be released as gaseous CO_2_ upon heating.^1^ Thus, LDHs have been extensively studied for their thermal decomposition behavior.

Numerous studies have reported significant variations in the gas evolution and structural transformation behaviors of LDHs depending on their metal ions. For example, in the case of Zn–Al–CO_3_ LDH, Kannan et al. observed a single-step weight loss at 523 K.^21^ They attributed the lower decomposition temperature of Zn–Al LDH to the strong tetrahedral site preference of the Zn^2+^ ion. Frost et al. reported that the thermal decomposition steps, breaking them down into four reactions: (1) release of some adsorbed water, (2) dehydroxylation of the metal hydroxide layers in two steps, (3) liberation of CO_2_ gas, and (4) CO_2_ evolution during the decomposition of compounds produced during the dehydroxylation of the hydrotalcite.^22,23^

In computational studies investigating the decomposition behavior of Zn–Al LDH, different interpretations have been reported. Alexandre et al. calculated differences in thermodynamic properties, such as enthalpy and entropy, in dehydrated reactions using ab initio simulations. They reported that the compound Zn_2/3_Al_1/3_(OH)_2_(CO_3_)_1/6_·4/6H_2_O dehydrates at approximately 448 K.^24^ They argue that when the interlayer water molecules are removed, the layered structure of LDH remains largely undestroyed, with its hexagonal lattice structure preserved. In contrast, Lombardo et al. used X-ray powder diffraction and molecular dynamics approaches, reporting that [Zn_0.65_Al_0.35_(OH)_2_] (CO_3_)_0.175_·0.69H_2_O cannot maintain the typical LDH structure when completely interlayer water is released (achieved at 453 K).^25^

Recently, Shimamura et al. conducted a characterization of the thermal decomposition behaviors of Zn–Al LDH using X-ray absorption near-edge spectroscopy (XANES). They reported that below 473 K, both Zn^2+^ and Al^3+^ ions maintain 6-fold coordination, even though the crystal structure of LDH becomes disordered at 473 K.^26^

In contrast, for Co–Al LDH, Marshall et al.^27^ identified two distinct regions of thermal decomposition behavior. The first region, occurring below 473 K, involves the removal of interlayer water molecules. The second region, at temperatures exceeding 473 K, is attributed to the dehydroxylation of metal hydroxide layers and the decomposition of interlayer CO_3_^2–^ ions. Velu et al. proposed that the oxidation of Co^2+^ to Co^3+^ occurs within a temperature range (approximately 533 K), coinciding with the endothermic decomposition of interlayer CO_3_^2–^ ions and the hydroxyl layer.^28^ Pérez-Ramírez et al. reported a three-step transformation for Co–Al LDH, encompassing the release of interlayer water, complete dehydroxylation, partial decomposition of the carbonates, and the removal of remaining carbonate groups.^29^ In a recent paper, Radha et al. reported that Co–Al LDH decomposes below 525 K, with the decomposition reaction preceded by the formation of an intermediate hydroxide, leading to aperiodic layers.^30^ This aperiodicity is modeled by randomly placing Co^2+^ ions in tetrahedral sites within the interlayer spaces. In addition, they reported that Co–Al LDH shows a two-step decomposition process in a nitrogen N_2_ atmosphere, while it undergoes a one-step decomposition in an air atmosphere.^31^ The effect of the atmosphere on decomposition behaviors was also reported by Khassin et al., who reported that the presence of nitric oxide (NO) in the gas phase decreases the decomposition rate of Co–Al LDH.^32^ Although numerous studies have investigated the thermal decomposition behavior, including gas evolution and structural transformation behaviors of LDHs at elevated temperatures, there have been inconsistencies in these studies and lack of detailed understanding of the influence of different metal ions in LDHs is still lacking. As described above, for *M*–Al LDH (M = Zn or Co) as well as Mg–Al LDH, the inconsistencies are found even in the number of transformation steps (2 or 3), when the layered structures collapses (after release of interlayer water molecules, dehydroxylation of layers, or decomposition of CO_3_^2–^ ions) and the effect of atmosphere. Thus, our study focuses on comprehending the effect of the choice of divalent metal ions (*M*^2+^) in LDHs.

In a previous study, we provided a comprehensive description of the structural transformation steps in well-crystallized Mg–Al LDH samples with an Mg/Al ratio of 2.^33^ Our analysis was accompanied by quantitative evidence,^33,34^ providing a clear understanding of these transformation steps: release of the interlayer water (Step (1)), partial dehydroxylation of the hydroxide layers followed by the of coordination of interlayer CO_3_^2–^ ions to the metals (Step (2)), and collapse of the layered structure accompanied by complete dehydroxylation of the layers, and the decomposition of interlayer CO_3_^2–^ ions, at a relatively high temperature (Step (3)). The Step (1) causes a large decrease in the LDH’s interlayer distance, decreasing it from approximately 7.6 to 6.7 Å.^33^ In addition, our research investigated into the multistep chemical and structural transformations of Mg–Al LDH particles with different Mg/Al ratios at elevated temperatures.^34^ Notably, the structural transformations observed in LDH particles with different Mg/Al ratios closely resemble those of well-crystallized Mg–Al LDH crystals.^33^ Despite some apparent differences, such as LDHs with Mg/Al ratios of 2 exhibiting a distinct three-step transformation and those with a ratio of 3 seemingly undergoing a two-step transformation, it is important to note that both cases essentially consisted of three essential steps. The difference is primarily due to differences in the temperature range of Step (2).

**Figure 1:**
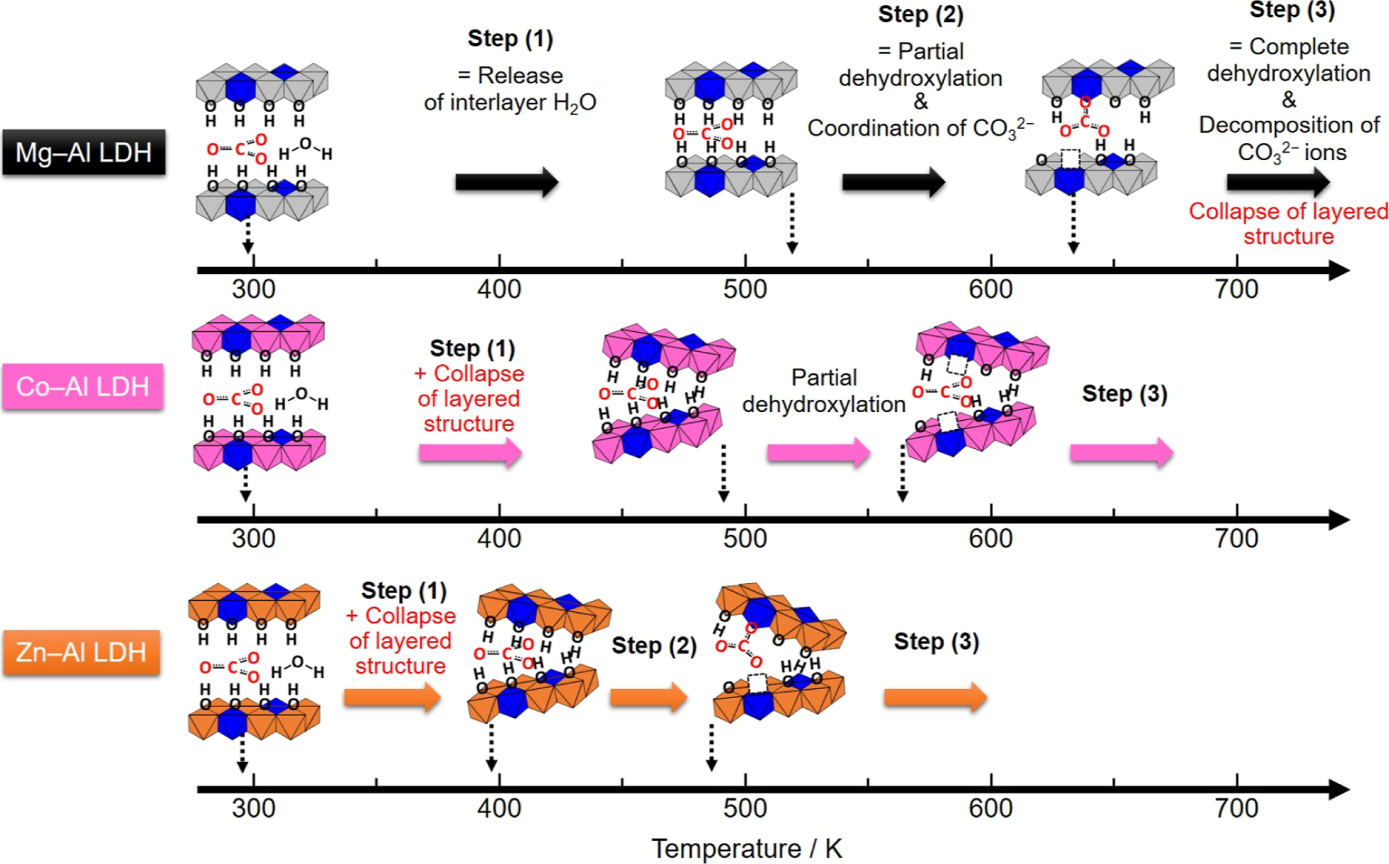
Schematic illustration of *M*–Al LDHs (M = Mg, Co, or Zn) structural/chemical transformation at elevated temperatures elucidated in this study. The metal hydroxide layers are drawn as edge-shared octahedra.

In this study, we placed our primary focus on comprehending how divalent metal ions in LDHs influence thermal decomposition behaviors. We analyzed the chemical and structural transformations for *M*–Al LDH samples, (M = Mg, Co, and Zn). We used samples with *M*/Al atomic ratio of 2. The advantage of this composition is simplicity of the structure: in the structure only one pattern of metal ion arrangement in the layers is possible, and all hydroxyl groups experience the same chemical environment: each hydroxyl group coordinates to two *M*^2+^ ions and one Al ion. Furthermore, our approach is built upon the clear-cut findings regarding Mg–Al LDH, as previously revealed in our previous studies.^33,34^ Moreover, our distinctive advantage lies in the quantitative analysis we employed, enabling us to determine the amounts of evolved H_2_O and CO_2_ during each reaction step at elevated temperatures. This quantification was achieved through the measurement of CO_2_ gas evolution combined with thermogravimetric analysis (TGA). Despite variations in reaction temperatures across the different steps, the chemical transformations observed for Zn–Al LDH and Co–Al LDH mirror those of Mg–Al LDH (as schematically summarized in Figure 1), encompassing three key steps: release of interlayer water (Step (1)), partial dehydroxylation of metal hydroxide layers, and complete dehydroxylation of layers and decomposition of interlayer CO_3_^2–^ (Step (3)). In the cases of Mg–Al LDH and Zn–Al LDH, the second step involved coordination of CO_3_^2–^ to metal ions (i.e., Step (2)). Furthermore, the structural transformations for each sample exhibit difference: The layered structure of Zn–Al LDH and Co–Al LDH collapsed in Steps (1), and that of Mg–Al LDH Step (3). In this study, we use the term “collapse of layered structure” to include disorder of layered structure that brings about disappearance of 0 0 *l* diffraction (Figure 1). The clear interpretation of the decomposition behavior for Mg–Al LDH, as established in our previous studies,^33,34^ combined with the quantitative analysis and in situ measurements, has enabled a detailed interpretation of the chemical and structural transformations in LDHs. Consequently, this paper provides valuable insights into the effect of divalent metal ions in LDHs on their thermal decomposition behavior.

## Methods

### Preparation of LDH Samples

We searched and selected from the literature the LDH preparation methods giving similar particles sizes. A powder sample of Mg–Al LDH was synthesized through a hydrothermal method.^35^ Mg(NO_3_)_2_·6H_2_O (2.308 g), Al(NO_3_)_3_·9H_2_O (1.688 g), and hexamethylenetetramine (1.640 g) were dissolved in 36 cm^3^ of deionized water. This solution was transferred into an autoclave (45 cm^3^) and subjected the autoclave to hydrothermal treatment for a duration of 24 h at 413 K. After cooling to room temperature, the resulted solids were filtered. We washed them thoroughly with deionized water. Subsequently, they were dried in vacuo at room temperature. To facilitate the exchange of anions with carbonate ions, we took the dried solid sample (0.2 g) and dispersed it in a NaHCO_3_ solution (1 mol dm^−3^, 200 cm^3^). We stirred the mixture for a duration of 12 h at room temperature. The resulted solids were filtered and washed with deionized water. Finally, they were dried in vacuo at room temperature. This process yields Mg–Al LDH powder with CO_3_^2–^ as interlayer anions. The Mg/Al ratio of this obtained sample was determined to be 2.03 through the use of ICP-AES.

Both Co–Al LDH and Zn–Al LDH were prepared using a homogeneous coprecipitation method.^36^*M*^2+^Cl_2_·6H_2_O (3 mmol, *M*^2+^ = Co^2+^ or Zn^2+^), AlCl_3_·6 H_2_O (1.5 mmol), and urea (10.5 mmol) were dissolved in 300 cm^3^ of deionized water. This solution was heated for 48 h at 370 K while maintaining continuous stirring. We then filtered the obtained product. Thoroughly washed the filtered product with deionized water and subsequently, washed the product with ethanol two times, repeating the washing process for each. Finally, we dried it in ambient atmospheric conditions. This process yields Co–Al LDH or Zn–Al LDH powder. To facilitate the exchange of anions with carbonate ions, the dried solid sample (0.2 g) was dispersed in a NaHCO_3_ solution (1 mol dm^−3^, 200 cm^3^). We stirred the mixture for a duration of 12 h at room temperature. Filtered the resulting solids and thoroughly washed the filtered solids with deionized water. Finally, we dried in vacuo at room temperature. This process yields Co–Al LDH and Zn–Al LDH powder with CO_3_^2–^ as interlayer anions. The Co/Al ratio for the Co–Al LDH sample and the Zn/Al ratio for the Zn–Al LDH sample were determined to be 1.77 and 1.97, respectively using ICP-AES.

### Characterization of Samples and In Situ Measurements to Trace Transformations

The *ex situ* XRD patterns were measured using a D2 PHASER diffractometer (Bruker) using Cu *K*α radiation. The morphologies of LDH sample particles were observed using a field emission scanning electron microscope (FE-SEM; Hitachi S-4800). Two modes of acceleration voltage were used: 5.0 and 0.5 kV (accelerated with 2.0 kV and retarded with 1.5 kV). The working distance between the sample and the objective lens was set as 1.5−2.0 mm. The lower voltage (0.5 kV) and short working distance are suitable for detailed observation of surface textures. Raman spectra were taken with an NRS-4500 spectrometer (JASCO Co. Ltd.) (532 nm laser of ∼1.9 mW). The rates of gaseous H_2_O and CO_2_ evolution from the LDH samples upon heating were measured using a gas-flow system equipped with a Q-mass spectrometer. The LDH sample (30 mg) was placed in a quartz tube reactor and heated to 1073 K at a temperature ramp rate of 10 K min^–1^ under an Ar flow (100 cm^3^ min^–1^). The outlet gas was analyzed by the Q-mass spectrometer (CO_2_, *m*/*z* = 44 and H_2_O, *m*/*z* = 18). Absolute CO_2_ evolution rates were determined using the Q-mass detector calibrated with a standard gas. Thermogravimetry-differential thermal analysis (TG-DTA) were carried out to obtain quantitative information regarding the desorbed gases during the heating of the samples. The LDH sample (10 mg) was placed in a platinum pan and heated to 1273 K at a temperature ramp rate of 10 K min^–1^ under an air flow (100 cm^3^ min^–1^). Variable-temperature powder XRD measurements were conducted under vacuum conditions (in situ XRD) using a D8 advance diffractometer (Bruker) equipped with an in situ sample chamber (Anton-Paar TTK-450). In situ Fourier transform infrared (FT-IR) adsorption spectroscopy was performed using an FT-IR 4200 spectrometer (JASCO). A glass cell connected to a vacuum system was used for these measurements.

### Estimation of Reaction Energies by First-Principles DFT Calculations

The structural models of hydroxide layer slabs were created and optimized using a combination of software package Materials Studio Visualizer (BIOVIA), and first-principles DFT calculation (CASTEP 2021), respectively. The unit cells of the periodic structured models included a single layer slab of metal hydroxides similar to those found in LDHs. The structural information for the layer was extracted from reported single-crystal experimental data.^37^ To eliminate interactions between layers, the interlayer distance was set to approximately 23 Å. The interlayer anions were omitted, and appropriate positive charges were introduced for the calculations. The structural optimization was performed with fixed cell parameters. A plane wave basis set cutoff of 630 eV was used for the calculations. Dehydroxylated metal hydroxide layer models were created by removing a pair of a hydroxyl (OH^–^) group and a proton (H^+^) from each of the models, and their structural optimization was performed. A model containing a single H_2_O molecule was placed at the center of a 20 × 20 × 20 Å cell. The structure of this H_2_O molecule was optimized to obtain its total energy. The total energy change (Δ*E*) for the dehydroxylation reaction was estimated by subtracting the sum of the total energies of the dehydroxylated and water molecule models from the total energy of the predehydroxylation model. Models representing metal hydroxide layers before and after the decomposition of monodentate CO_3_^2–^ coordinating to the hydroxide layer were created. A 2 × 2 supercell was constructed in the *a* and *b* directions, and dehydroxylated the model. A CO_3_^2–^ ion was placed on the coordinatively unsaturated site generated by dehydroxylation on the slab model. The structure of the entire model was optimized to create the predecomposition model. Furthermore, the after-decomposition model was created by removing two O atoms and one C atom from the CO_3_^2–^ in the predecomposition model. The structure of the after-decomposition model was also optimized. A model containing a single CO_2_ molecule was placed at the center of a 20 × 20 × 20 Å cell. The structure of this CO_2_ molecule was optimized to obtain its total energy. The total energy change (Δ*E*) for the decomposition reaction of CO_3_^2–^ was estimated by subtracting the sum of the total energies of the after-decomposition and carbon dioxide models from that of the predecomposition model.

## Results and Discussion

### Preliminary Theoretical Estimations of Reaction Energies by First-Principles DFT Calculation

**Figure 2:**
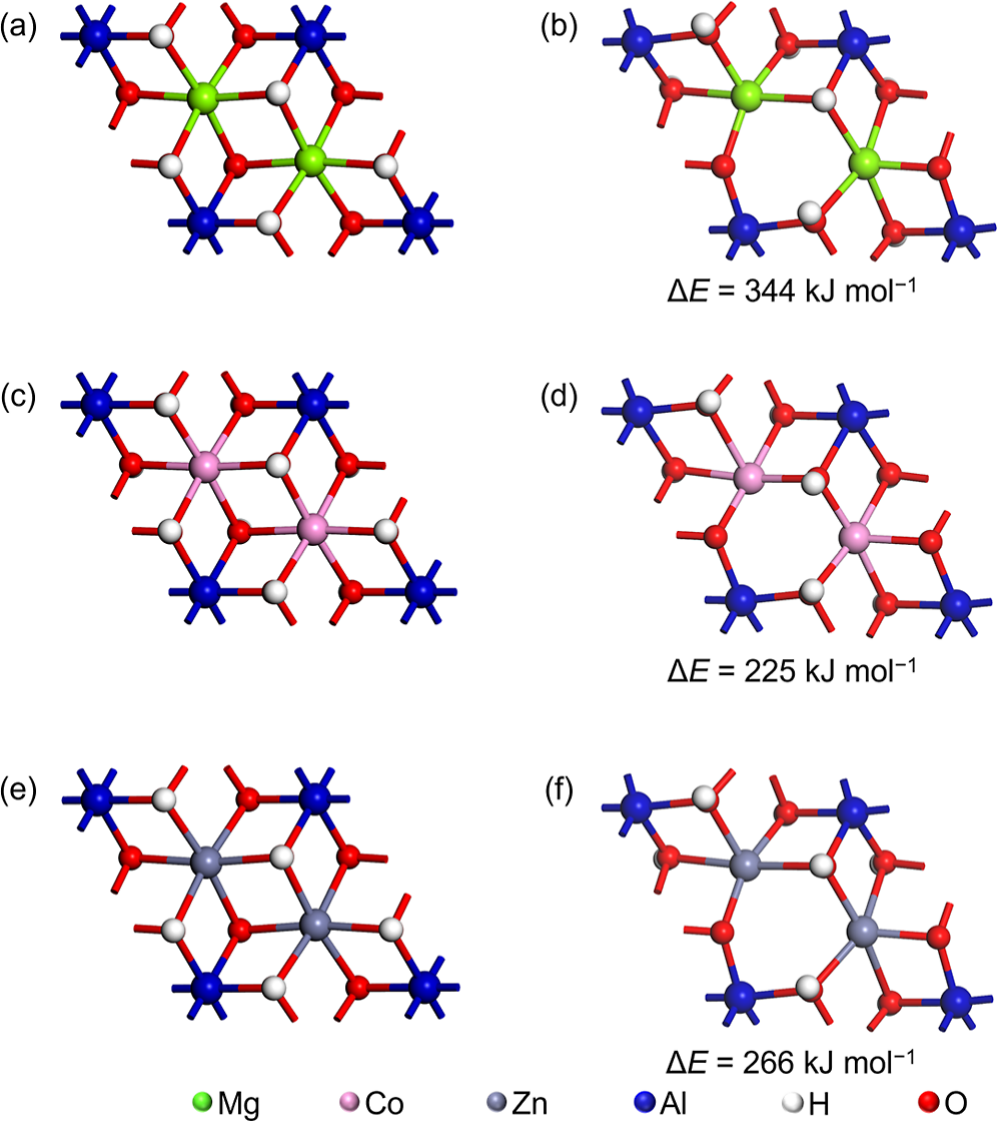
Structurally optimized models before (left) and after (right) partial dehydroxylation reaction for LDH slabs. (a,b) Mg–Al LDH slab models, (c,d) Co–Al LDH slab models, (e,f) Zn–Al LDH slab models. Values of Δ*E*s are the calculated reaction energies of dehydroxylation.

To obtain preliminary insights into the effect of various divalent metal ions on the multistep transformation of LDHs, we conducted calculations to estimate the reaction energies associated with two key processes: the dehydroxylation of metal hydroxide layers and the decomposition of CO_3_^2–^ ions coordinated to these layers in various LDHs. In a previous study,^33^ we proved that within an intermediate temperature range (approximately 580 K), the metal hydroxide layers of well-crystallized Mg–Al LDH large crystals (Mg/Al = 2) experienced partial dehydroxylation, followed by coordination of interlayer CO_3_^2–^ anions to the metal ions. As the temperature increased (approximately 640 K), complete dehydroxylation of the metal hydroxide layers and decomposition of CO_3_^2–^ took place. Therefore, understanding the reaction energies associated with dehydroxylation and CO_3_^2–^ decomposition is important for gaining insights into the multistep chemical and structural transformation behaviors. In this study, we employed hydroxide layer slab models featuring different divalent metal ions (Mg^2+^, Co^2+^, and Zn^2+^) to calculate these reaction energies. Figure 2 shows the structurally optimized models before and after partial dehydroxylation reactions for LDH slabs of the three LDH types, along with the calculated energies for dehydroxylation reactions. Notably, the order of reaction energies was as follows: Co–Al LDH (225 kJ mol^–1^) < Zn–Al LDH (266 kJ mol^–1^) < Mg–Al LDH (344 kJ mol^–1^).

**Figure 3:**
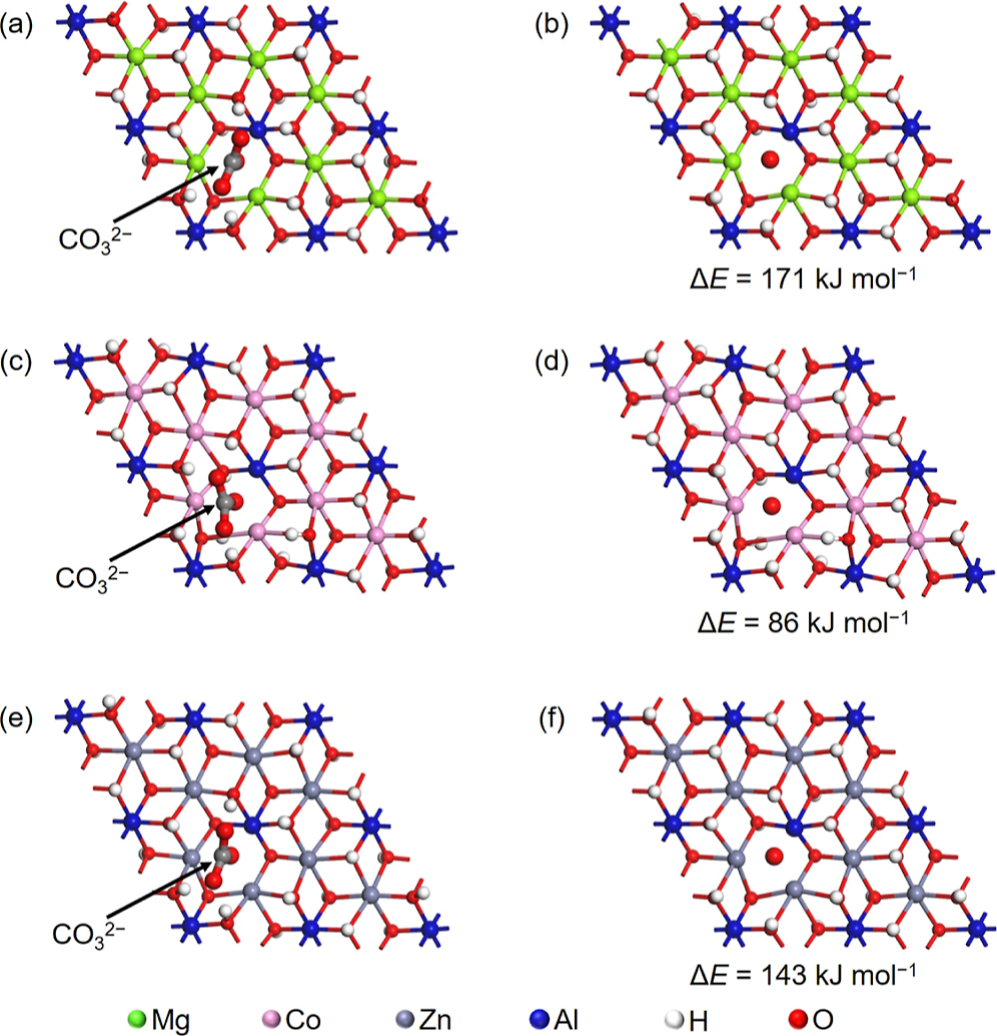
Structurally optimized models before (left) and after (right) decomposition reaction of CO_3_^2–^ coordinating to the metal hydroxide layer for LDH slabs. (a,b) Mg–Al LDH slab models, (c,d) Co–Al LDH slab models, (e, f) Zn–Al LDH slab models. Values of Δ*E*s are the calculated reaction energies of the decomposition reaction of CO_3_^2–^.

Figure 3 shows the structurally optimized models before and after the decomposition of CO_3_^2–^ ions coordinated to the metal hydroxide layer slabs of LDHs, alongside the calculated reaction energies for CO_3_^2–^ decomposition. Remarkably, the order of decomposition reaction energies aligns with that of dehydroxylation energies: Co–Al LDH (86 kJ mol^–1^) < Zn–Al LDH (143 kJ mol^–1^) < Mg–Al LDH (171 kJ mol^–1^). Consequently, it is expected that the order of temperature ranges where these chemical transformations occur will follow the same order: Co–Al LDH < Zn–Al LDH < Mg–Al LDH, both for dehydroxylation and CO_3_^2–^ decomposition reactions. Here it should be noted that the present calculation adopts a simple assumption to extract the effect of the different nature of divalent cations: we assume that layered LDH slab structure is maintained throughout the reactions. This point will be discussed in a later section.

### Characterization of LDH Samples

**Figure 4:**
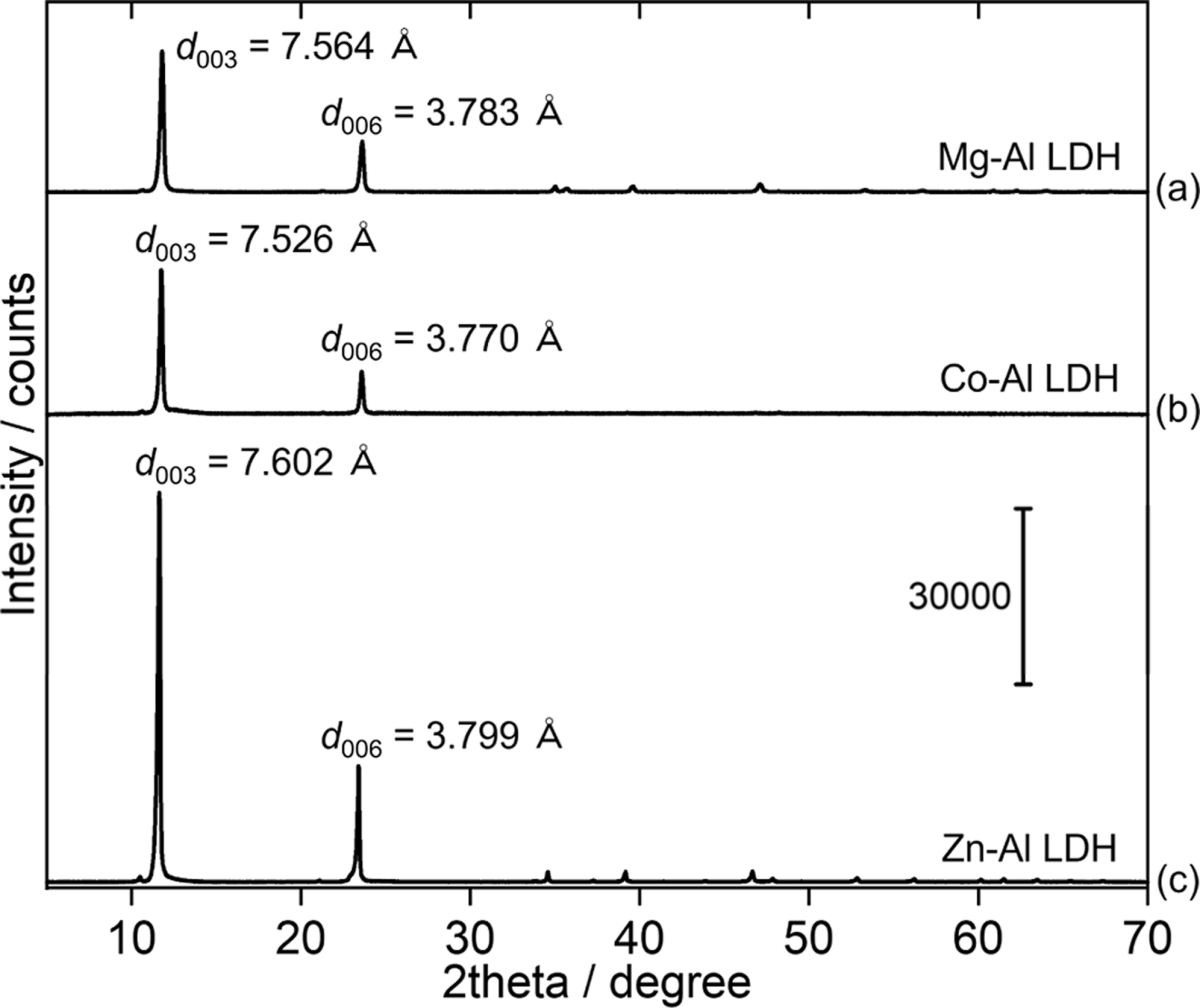
XRD patterns of LDH samples. (a) Mg–Al LDH. (b) Co–Al LDH. (c) Zn–Al LDH.

**Figure 5:**
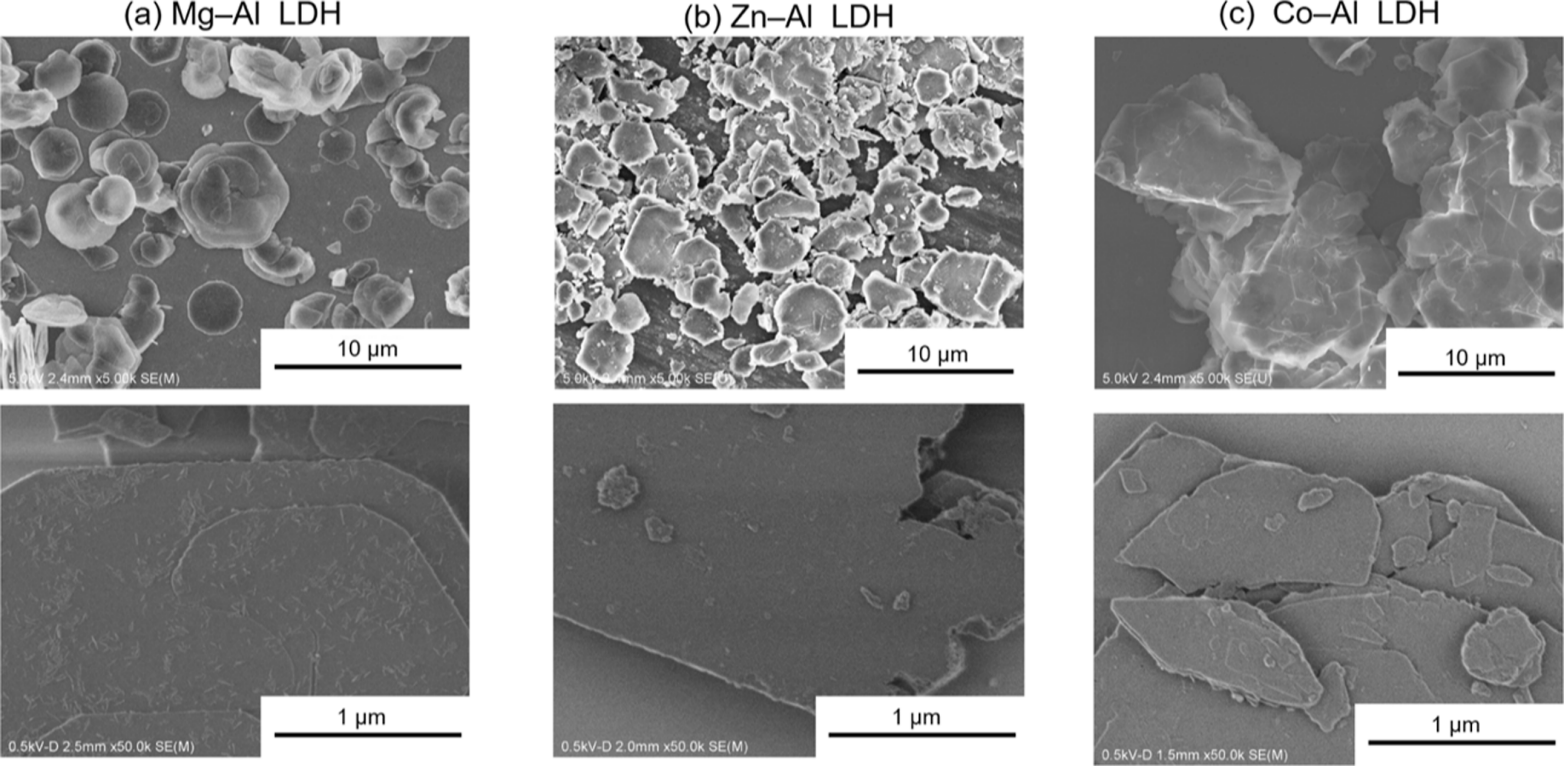
SEM images of LDH samples. (a) Mg–Al LDH. (b) Zn–Al LDH. (c) Co–Al LDH. The top and bottom panels are images taken with acceleration voltages of 5.0 and 0.5 kV, respectively.

Next, we performed a fundamental characterization of three distinct LDH samples: Mg–Al LDH, Zn–Al LDH, and Co–Al LDH. Our investigation began with the examination of ex situ XRD patterns, presented in Figures 4 and S1 in the Supporting Information, revealing the characteristic diffraction peaks indicative of crystalline LDHs. These peaks, located at approximately 12 and 22°, were assigned to the basal planes 003 and 006 within their layered crystal structures. The interplanar distances (*d*_003_) appeared to be similar for all three samples, irrespective of the variation in divalent cations (Mg^2+^, Co^2+^, and Zn^2+^). Peaks at higher angles in Figure 4b seem to be missing, possibly due to preferred orientation of the sample particles. As shown in Figure 5, the SEM images demonstrate the formation of plate-like particles with sizes of several μm for all three samples. Surface textual images taken with a low acceleration voltage (the bottom panels in Figures 5 and S2–S4 in the Supporting Information) for Co–Al and Zn–Al LDHs showed very smooth surfaces of the particles and no trace of minor components. In the surface image of Mg–Al LDH, a very small amount of fibrous substance was detected on the LDH particles surfaces. Judging from the very small volume fraction of the substance, of which effect on the thermal structural transformation behaviors might be negligible.

Raman spectra for the three samples are shown in Figure S5 in the Supporting Information. These spectra indicate high purity of the three samples. All signals detected for Mg–Al LDH (Figure S5a) are completely identical with those reported for well-crystallized quintinite Mg_4_Al(OH)_12_CO_3_·3 H_2_O (the bar chart in Figure S5).^38^ The spectrum of Co–Al LDH (Figure S5b) is identical with that reported in the literature.^39^ There is no trace of impurity in the spectrum of Zn–Al LDH (Figure S5c). These results demonstrate high purity of the samples.

### Multistep Structural/Chemical Transformation of Mg–Al LDH at Elevated Temperatures

Next, we examined the structural and chemical transformation behavior of the Mg–Al LDH sample, a subject that has undergone extensive analysis by researchers,^37,40−44^ including our own prior work.^19,33,34^ We have previously reported a comprehensive analysis of the Mg–Al LDH behavior, offering atomic and molecular-level pictures for each step of the transformation.^33,34^ Consequently, we considered Mg–Al LDH a suitable standard for comparative purposes when investigating LDHs consisting of different ions such as Zn^2+^ and Co^2+^.

**Figure 6:**
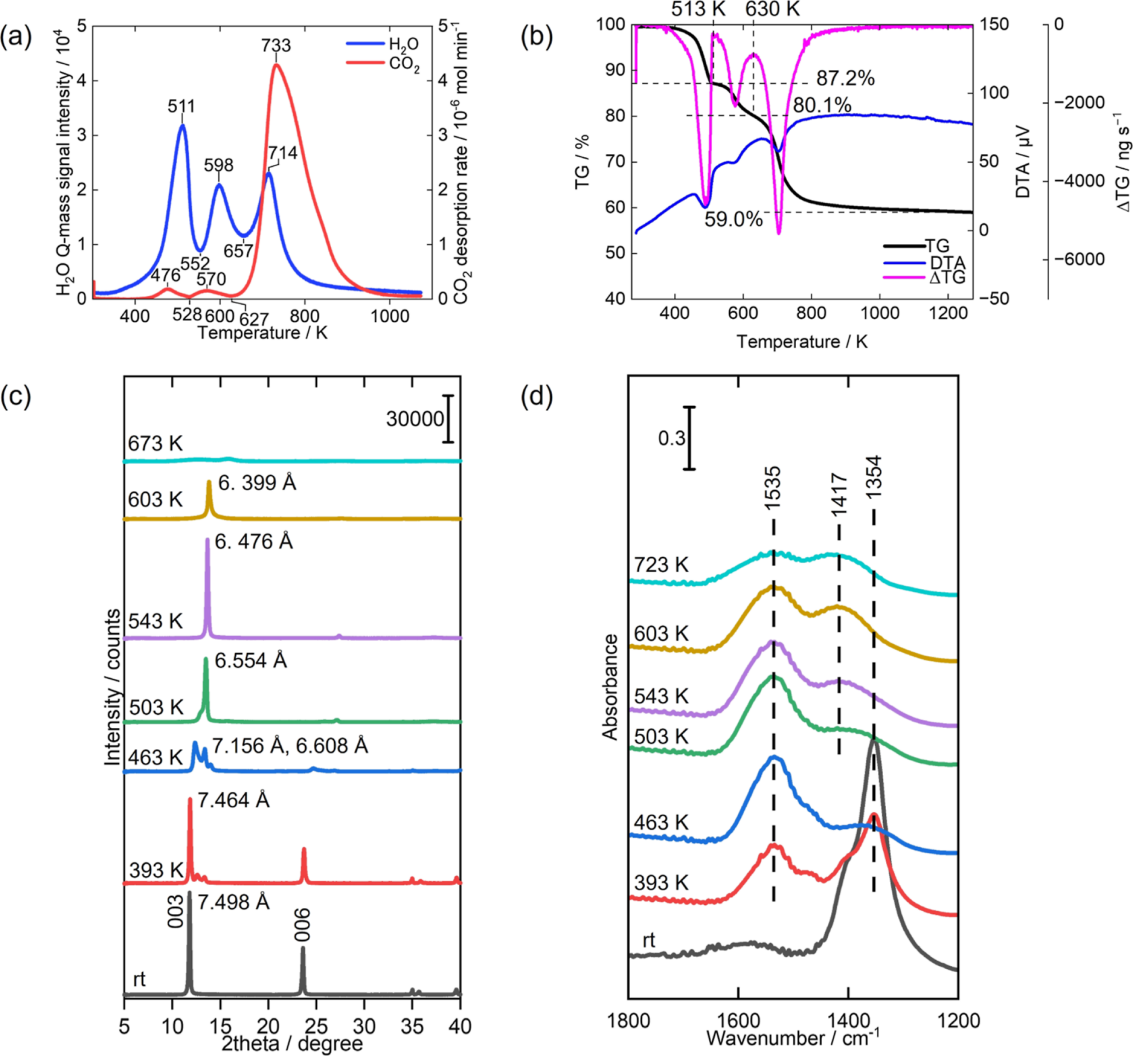
Results of various measurements for Mg–Al LDH. (a) Evolution rates of gaseous H_2_O and CO_2_ under continuous heating, (b) TG-DTA-DTG curves, (c) in situ XRD patterns in vacuo at elevated temperatures, (d) in situ FT-IR spectra in vacuo at elevated temperatures.

Figure 6 shows evolution rates of gaseous H_2_O and CO_2_, TG-DTA curves, in situ XRD patterns, and in situ FT-IR spectra for the Mg–Al LDH sample subjected to elevated temperatures. A notable advantage of our experimental approach lies in our ability to quantify the absolute amounts of released CO_2_. We achieved this quantification by integrating the CO_2_ gas evolution rates for each step of the multistep chemical transformation depicted in Figure 6a. Detailed results are listed in Table 1. As highlighted in our previous studies,^33,34^ the chemical and structural transformations occur in three distinct steps: the release of interlayer H_2_O molecules (Step (1)), partial dehydroxylation of the hydroxide layers accompanied by the formation of coordinatively unsaturated sites and the coordination of interlayer CO_3_^2–^ anions to the metals (Step (2)), and ultimately, complete dehydroxylation coupled with a substantial release of CO_2_ (Step (3)), culminating in the collapse of the layered structure.

**Table 1:** CO_2_ Amounts Released During Multi-step Transformation of LDHs Determined from Gas Evolution Rates

sample	first step		second step		third step		higher temp.	
	temp./K	CO_2_ evolution/mmol g–1	temp./K	CO_2_ evolution/mmol g–1	temp./K	CO_2_ evolution/mmol g–1	temp./K	CO_2_ evolution/mmol g–1
Mg–Al LDH	416–528	0.027	528–627	0.026	627–1078	1.6	1078–	trace
Zn–Al LDH	400–495	0.022	495–533	0.040	533–763	0.47	763–	0.34
Co–Al LDH	408–520	0.045	520–551	trace	551–731	0.64	731–	0.28

The results obtained for the Mg–Al LDH sample in this study (Figure 6) consistently align with the three-step transformations previously analyzed in our previous study.^33,34^ Particle size difference seems to cause small differences in gas evolution behaviors between the Mg–Al LDH sample in the present study (ca. 3–8 μm in size) and that in the previous report (ca. 60–100 nm in size).^34^ A substantial release of H_2_O was detected at approximately 511 K in the H_2_O evolution curves (Figure 6a). Concurrently, a corresponding endothermic weight loss of significance was observed in the TG-DTA curves over the temperature range of room temperature to 513 K (Figure 6b). This pronounced evolution of H_2_O is due to the release of interlayer H_2_O, which constitutes Step (1) of the transformation process. In situ FT-IR results further corroborate the release of interlayer H_2_O (Figure 6d). At room temperature, a strong peak at 1354 cm^–1^ was observed, corresponding to the asymmetric stretch vibration of interlayer CO_3_^2–^ ions hydrogen-bonded to interlayer H_2_O molecules. In Step (1), as interlayer H_2_O molecules were released, the hydrogen bonds between interlayer CO_3_^2–^ and H_2_O molecules were disrupted. Accordingly, at 543 K, the peak at 1354 cm^–1^ almost disappeared, replaced by a new peak at 1535 cm^–1^ (Figure 6d). This shift to a higher wavenumber is indicative of the loss of hydrogen bonds. Moreover, the in situ XRD patterns (Figure 6c) reflect the release of interlayer H_2_O molecules (Step (1)), as evident by a significant decrease of approximately 0.9 Å in the interlayer distance, transitioning from 7.5 to 6.6 Å. In our previous study, we revealed that the underlying rationale for this large decrease, which can be attributed to the wave-like shape of the metal hydroxide layers.^34^

Moving to Step (2), a certain amount of H_2_O evolved at 598 K (Figure 6a), aligning with the partial dehydroxylation of the metal hydroxide layers. This partial dehydroxylation is mirrored in the TG curve (Figure 6b) as a weight loss occurring in the 513–630 K temperature range. Moreover, during the temperature range of 603 K in the in situ FT-IR spectrum (Figure 6d), two distinct split peaks emerged at 1535 and 1417 cm^–1^. These split peaks are assigned to monodentate CO_3_^2–^ ions coordinating with metal ions.^33,34^ In other words, the coordination of interlayer CO_3_^2–^ ions occurred at the coordinatively unsaturated sites generated through the partial dehydroxylation of the metal hydroxide layers. Simultaneously, in situ XRD patterns showed a small decrease in interlayer distance, diminishing from 6.6 to 6.4 Å (503–603 K in Figure 6c). This gradual change in the interlayer distance corresponds to the ongoing chemical transformation described above, with further details available in our previous study.^34^

Finally, in Step (3), a substantial release of both CO_2_ and H_2_O occurred at approximately 720 K, because of the decomposition of interlayer CO_3_^2–^ ions and the complete dehydroxylation of metal hydroxide layers (Figure 6a). Accordingly, a large weight loss was observed within the 630–900 K temperature range in the TG curve (Figure 6b). The in situ FT-IR spectra (Figure 6d) demonstrated a significant reduction in peak intensities at 1535 and 1417 cm^–1^ at 723 K, aligning with the aforementioned decomposition of CO_3_^2–^ ions. Moreover, the in situ XRD patterns depicted the collapse of the layered structure, with the diffraction peaks disappearing at 673 K (Figure 6c).

These results for the Mg–Al LDH sample in this study (Figure 6) align with the molecular-level pictures of the structural/chemical three-step transformation previously described in our study.^33,34^

### Analysis of Structural/Chemical Transformation of Zn–Al LDH at Elevated Temperatures

To further investigate the effect of different metal cations, we proceeded to analyze the chemical transformation behavior of Zn–Al LDH, referencing the interpretation of Mg–Al LDH described above. A significant difference is that Zn–Al LDH lost its layered structure immediately after the release of most interlayer H_2_O molecules.

**Figure 7:**
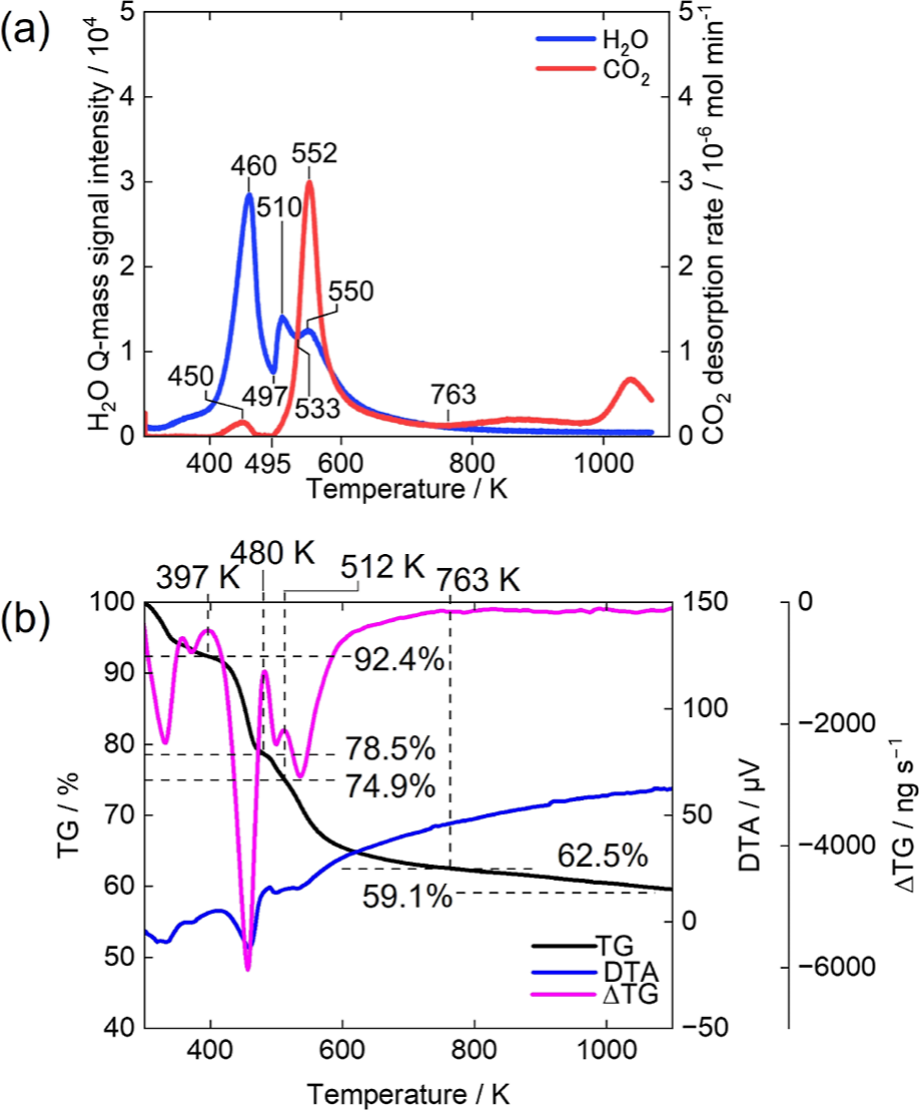
TG Analysis and gas evolution behavior of Zn–Al LDH during continuous heating. (a) Evolution rates of H_2_O and CO_2_, (b) TG-DTA-DTG curves.

Figure 7a shows the evolution rates of gaseous H_2_O and CO_2_ upon heating for Zn–Al LDH. The gas evolution profile clearly showed distinct three-step transformation. Initially, a large amount of H_2_O was released at 460 K, accompanied by a small amount of CO_2_ release at 450 K. In the next step, at around 510 K, a certain amount of H_2_O was released. CO_2_ release commenced at 495 K, but in smaller amounts. In a higher temperature range, H_2_O began to release at 550 K, concomitant with a significant CO_2_ emission at 552 K. Finally, at higher temperatures (763–1073 K), a continuous, but small, release of CO_2_ persisted. We quantified the amount of CO_2_ evolved in each step by integrating the gas evolution rate in Figure 7a, with the values summarized in Table 1.

Figure 7b shows the TG, DTA, and DTG curves for Zn–Al LDH. The TG curve shows weight losses in four temperature ranges: rt–397, 397–480, 480–512, and 512 K–. These temperature ranges align well with the three-step weight losses observed in the gas evolution (Figure 7a).

The small amount of CO_2_ evolution at 460 K is attributed to adsorbed carbonate species on the outer surfaces of the LDH sample (Figure 7a and Table 1). The CO_2_ release at temperatures above 763 K implicates that a small amount of CO_3_^2–^ remained despite the collapse of the layered structure. It is likely that small amounts of metal carbonates were formed and persisted up to 1073 K, as indicated by the CO_2_ release peak at approximately 1040 K (Figure 7a). Thus, the subsequent focus is on elucidating the chemical transformations in the three temperature ranges: approximately 397–480, 480–512, and 512–763 K, referred to as the first, second, and third steps, respectively.

**Figure 8:**
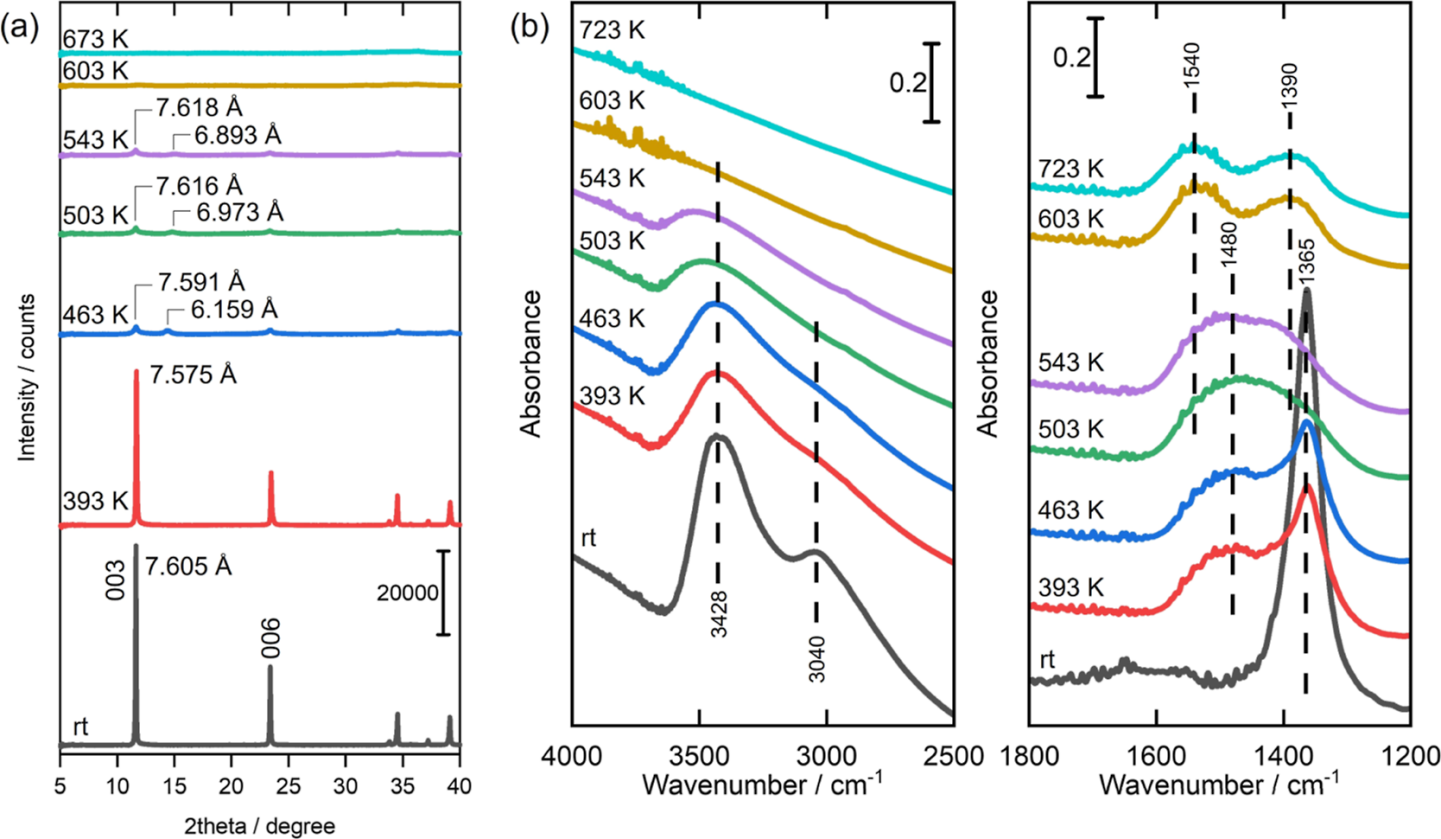
In situ measurement results for Zn–Al LDH in vacuum at elevated temperatures. (a) XRD patterns, (b) FT-IR spectra.

Subsequently, we focus on the structural transformation of CO_3_^2–^ in Zn–Al LDH, based on in situ experiments at elevated temperatures (Figure 8). The left panel of Figure 8b presents in situ FT-IR peaks assigned to OH stretching bands. The chemical environment of interlayer CO_3_^2–^ can serve as a sensitive probe into the structure of the compound, reflected in the spectra in the right panel of Figure 8b.

Let us discuss the transformation in the first step. The FT-IR spectrum at rt shows peaks at 3428 and 3040 cm^–1^. The peak at 3428 cm^–1^ corresponds to the OH stretching band, while the peak at 3040 cm^–1^ indicates a bridging mode involving interlayer CO_3_^2–^ and H_2_O molecules.^44^ The signal at 3040 cm^–1^ almost disappears upon heating to 463 K due to a reduction in interlayer H_2_O. Accordingly, a significant decrease is observed in the signal corresponding to interlayer CO_3_^2–^ hydrogen-bonding to water, and a new signal emerges at 1480 cm^–1^ at 393 and 463 K. Considering the disappearance of the signal at 3040 cm^–1^ and referring to the interpretation of Mg–Al LDH behavior, it is reasonable to attribute the new signal at 1480 cm^–1^ to CO_3_^2–^ that has lost hydrogen bonds with water. In the spectrum at 463 K, the peak at 1365 cm^–1^ decreases but still remains, indicating that a small amount of interlayer H_2_O molecules persist and continue to hydrogen-bond with interlayer CO_3_^2–^.

In situ XRD results (Figure 8a) reflect a significant difference between Zn–Al LDH and Mg–Al LDH: the peak at 7.6 Å, corresponding to the layered structure of Zn–Al LDH, almost disappears at 463 K. However, as indicated by in situ FT-IR, a small amount of interlayer H_2_O molecules still forms hydrogen bonds with CO_3_^2–^. These results suggest that the highly ordered layered structure of Zn–Al LDH disappears at 463 K, even before the complete elimination of H_2_O molecules.

Regarding the second step, the spectrum at 503 K indicates two important observations: interlayer H_2_O molecules are completely eliminated, and the hydroxide layers commence dehydroxylation. The disappearance of the peak at 1365 cm^–1^ confirms the first observation, while the appearance of a broad peak at approximately 1480 cm^–1^ in the spectrum at 503 K (Figure 8b) can be separated into three components at 1540, 1480, and 1390 cm^–1^ (as shown in Figure S6 in the Supporting Information). Based on the interpretation of Mg–Al LDH, the two newly appeared peaks (1540 and 1390 cm^–1^) are indicative of monodentate CO_3_^2–^ coordinating with metal ions. Therefore, the chemical transformation of Zn–Al LDH in the second step, around 503 K, appears similar to that in Step (2) for Mg–Al LDH: partial dehydroxylation of the hydroxide layers followed by CO_3_^2–^ coordination to metal ions. Looking back at the spectra at lower temperatures, the peak at 1480 cm^–1^ also appears somewhat broad at 393 and 463 K. Attempting curve fitting to separate the peak into three components did not yield reliable results. Given the presence of residual interlayer H_2_O molecules at 463 K, it seems unlikely that partial dehydroxylation of the layers proceeds within that temperature range. This point will be discussed in a later section.

The third step for Zn–Al LDH closely resembles Step (3) for Mg–Al LDH, as discussed below. In the higher temperature range of 480–512 K, the FT-IR peaks at 1540 and 1390 cm^–1^ show the presence of monodentate CO_3_^2–^ coordinating with metal sites.^33,34^ As the temperature increases from 543 to 723 K, the peak at 1480 cm^–1^ gradually decreases. Simultaneously, the peaks of monodentate CO_3_^2–^ exhibit slight an increase and then decrease (503–723 K). Furthermore, the intensity of the peak at 3428 cm^–1^ (OH stretching) decreases and finally disappears between 503 and 723 K, confirming the complete dehydroxylation of the metal hydroxide layers. Thus, in the third step of Zn–Al LDH, the hydroxide layers undergo complete dehydroxylation, leading to the decomposition of most interlayer CO_3_^2–^ and the release of a large amount of gaseous CO_2_. To summarize the case of Zn–Al LDH, the transformation occurs in three distinct steps, essentially mirroring Steps (1–3) in Mg–Al LDH. This point is an important novel insight found in this study.

The key difference between Zn–Al LDH and Mg–Al LDH is the disappearance of the layered structure just after Step (1) for Zn–Al LDH. Regarding this point, similar observations have been reported by Thomas et al.,^21^ Lombardo et al.^25^ and Shimamura et al.^26^ Shimamura et al. reported that the loss of the layered structure in Zn–Al LDH is due to the strong tetrahedral site preference of the Zn^2+^ ion. Shimamura et al. presented XANES spectra that indicated Zn^2+^ maintains 6-fold coordination at 473 K, despite the layered structure becoming disordered at this temperature.^26^ At this temperature Zn–Al LDH lost just interlayer H_2_O molecules, and the XRD peaks were significantly weakened and broadened at this temperature.^26^ The XANES spectrum indicated that heating to 673 K brought about changes of coordination environment of Zn^2+^ ions.^26^ Thus, their XANES observations are also consistent with our interpretation of Steps (1) and (2) of Zn–Al LDH. Lombardo et al. argued that the release of volatile water causes fine cracking along and across the layers.^25^

### Analysis of Structural/Chemical Transformation of Co–Al LDH at Elevated Temperatures

Moving on to our investigation of the decomposition behaviors of Co–Al LDH, let us summarize the results for Co–Al LDH. The transformation occurs in three steps, although the temperature range for each step differs from that of Mg–Al LDH or Zn–Al LDH. The chemical reactions in each step were interpreted similarly to those for Mg–Al LDH and Zn–Al LDH, except the behaviors of CO_3_^2–^ ions in the second step. Co–Al LDH loses the layered structure during the release process of interlayer H_2_O molecules which results in a different structural transformation from that of Mg–Al LDH. We will provide a more detailed explanation in later sections.

**Figure 9:**
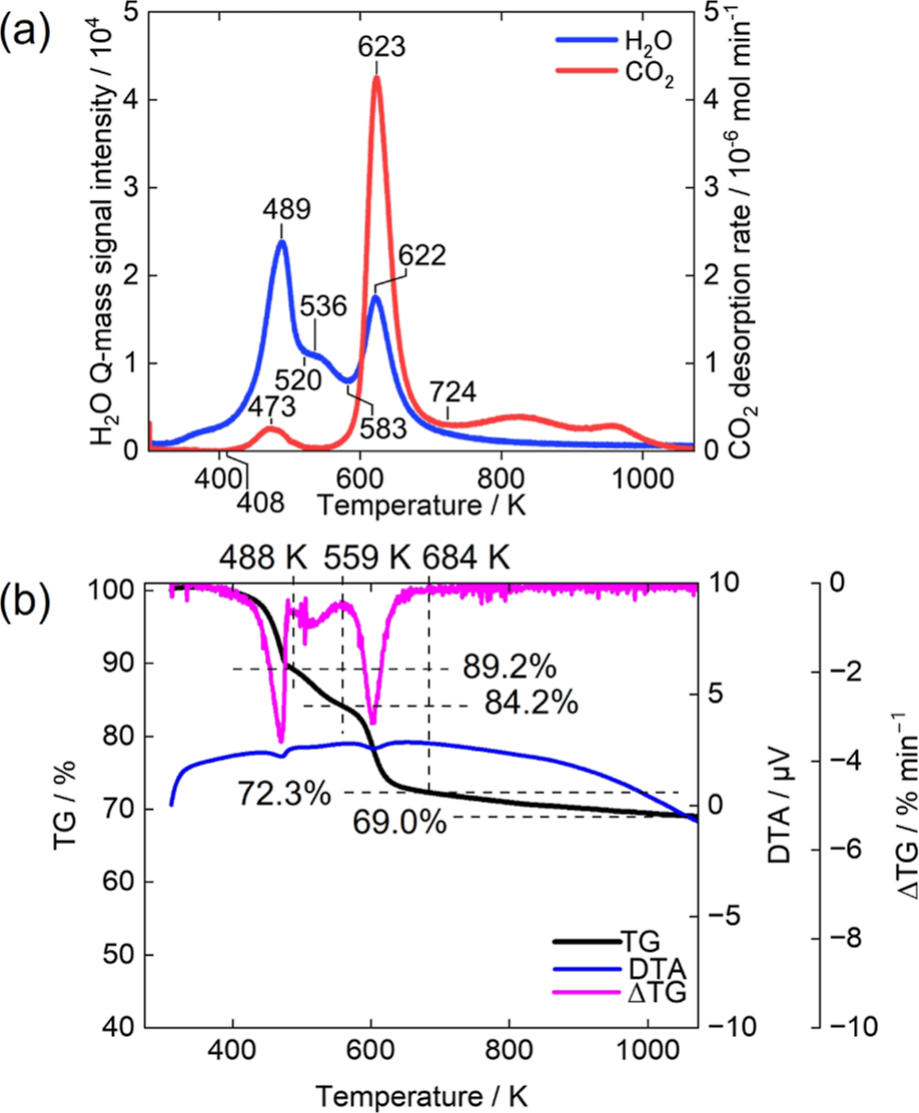
TG Analysis and gas evolution behavior of Co–Al LDH during continuous heating. (a) Evolution rates of H_2_O and CO_2_, (b) TG-DTA-DTG curves under He flow.

Co–Al LDH exhibits the release of gaseous H_2_O and CO_2_ in three distinct steps. Figure 9a shows the evolution rates of gaseous H_2_O and CO_2_ upon heating for Co–Al LDH. The gas evolution profile shows a three-step transformation. In the first step, a large amount of H_2_O is released at 489 K, along with a small amount of CO_2_ at 473 K. In the second step, a certain amount of H_2_O and trace amounts of CO_2_ are released around 536 K. In the third step, a large amount of H_2_O is released at 622 K, accompanied by a significant amount of CO_2_ at 623 K. Finally, at higher temperatures (724 K−), small amounts of H_2_O and CO_2_ are released continuously. We quantified the CO_2_ evolution amounts in each step by integrating the gas evolution rate in Figure 9a, and the values are shown in Table 1. The small release of CO_2_ at 473 K can be attributed to carbonate species adsorbed on the outer surfaces of the LDH sample (Figure 9a and Table 1). The CO_2_ release in the temperature range above 724 K implicates that a small amount of CO_3_^2–^ remains even though the metal hydroxide layers have already decomposed. It is likely that a small amount of metal carbonates forms and decomposes to release CO_2_ continuously. These results reveal that almost all H_2_O and CO_2_ are released in three steps, with a certain amount of CO_2_ being released continuously at higher temperatures for Co–Al LDH, as well as for Zn–Al LDH.

Figure 9b shows the TG, DTA, and DTG curves for Co–Al LDH. In the case of air atmosphere measurement, which is conducted under the same conditions as for Mg–Al LDH and Zn–Al LDH, Co–Al LDH shows a two-step weight loss pattern (Figure S7 in the Supporting Information). This result is inconsistent with the H_2_O and CO_2_ evolution rates measured in an Ar atmosphere (Figure 9a). A similar result had been previously reported,^31^ which was likely affected by the oxidation of Co^2+^. To address this discrepancy, a TGA measurement for Co–Al LDH was made in a He atmosphere using another apparatus (Figure 9b). The TG curve shows three-step weight losses occurring in the temperature ranges of room temperature–488, 488–559, and 559–1073 K. These temperature ranges for the three-step weight losses in the TG curve correspond well with those observed for the three steps in the gas evolutions (Figure 9a).

**Figure 10:**
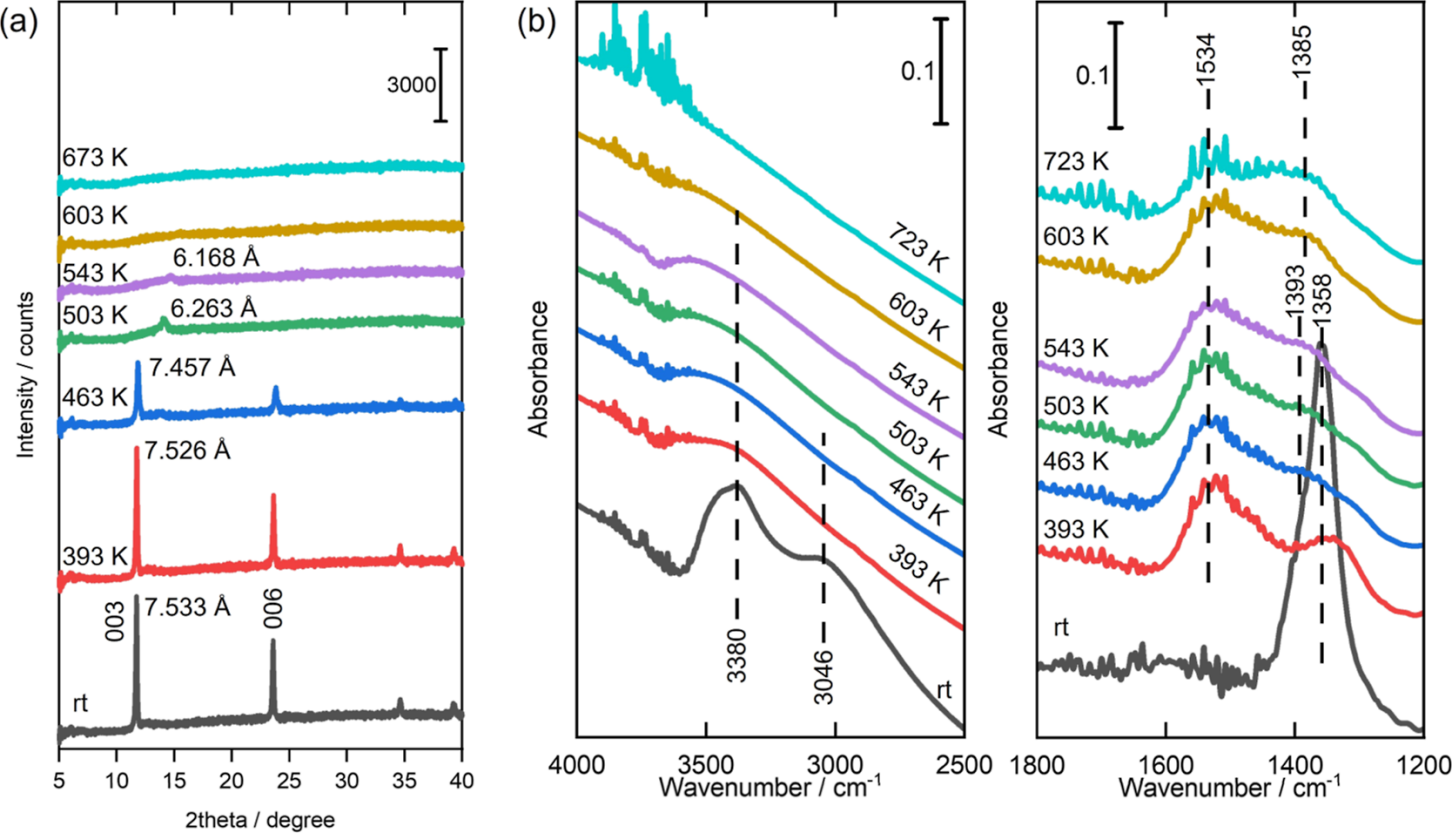
In situ measurement results for Co–Al LDH in vacuum at elevated temperatures. (a) XRD patterns, (b) FT-IR spectra.

Next, we will examine the structural transformation and the chemical environment of CO_3_^2–^ for Co–Al LDH. We investigated the crystal structure transformation through in situ XRD measurements at elevated temperatures (Figure 10a). In addition, we collected in situ FT-IR spectra to obtain information about the structures and states of interlayer CO_3_^2–^ during the thermal decomposition of Co–Al LDH (Figure 10b).

Initially, we observed that the crystallinity of Co–Al LDH decreased due to the release of interlayer H_2_O molecules in the first step. As mentioned previously, a large amount of H_2_O was released at approximately 490 K. Inside the temperature range, the in situ FT-IR spectra showed notable changes. Specifically, the peak at 3046 cm^–1^ almost disappeared, and the peak intensity at 3380 cm^–1^ decreased as the temperature increased from room temperature to 463 K (Figure 10b). These peaks assigned to OH stretching band, with the peak at 3040 cm^–1^ being interpreted as a bridging mode involving interlayer CO_3_^2–^ and H_2_O molecules.^44^ Thus, it is likely that the release of interlayer H_2_O molecules caused these changes in the FT-IR spectra. At 463 K, the interlayer CO_3_^2–^ lost its hydrogen-bonding with the interlayer molecules, resulting in the almost complete disappearance of the peak at 1358 cm^–1^ and the appearance of a new peak at 1534 cm^–1^. Simultaneously, the in situ XRD pattern showed the 003 peak with significantly reduced intensity (Figure 10a). At 503 K the 003 peak completely disappeared. Thus, Co–Al LDH lost its layered structure when it released the interlayer water.

In the XRD pattern at 503 K in Figure 10a, a very small peak for *d* = 6.263 Å was observed. In our previous study of Mg–Al LDH, the interlayer distance decreased by ca. 0.9 Å when it lost the interlayer water. Considering this, the small peak is likely due to Co–Al LDH after release of the interlayer water. The fact that the peak was very small also supports the near collapse of the layered structure when the interlayer water was just released. Here it should be noted that we use the term “collapse of layered structure” to include disorder of layered structure that brings about disappearance of 0 0 *l* diffraction as drawn in Figure 1, because the gas evolution quantitative analysis demonstrated that dehydroxylation of the hydroxide layers does not occur at this stage, as will be discussed in a later section.

In the subsequent second step, the metal hydroxide layers underwent partial dehydroxylation in the temperature range of 488–559 K. The in situ FT-IR spectra did not show significant transformations despite the temperature increase from 463 to 543 K. Thus, the changes in the coordination state of CO_3_^2–^ in the second step could not be clearly determined from the in situ FT-IR data.

In the final third step, large amounts of H_2_O and CO_2_ are released by complete dehydroxylation of the metal hydroxide layers and the decomposition of CO_3_^2–^ within the temperature range of 559–684 K. During this step, the in situ FT-IR spectra show the disappearance of the peak at 3380 cm^–1^ at 603 K, signifying the complete dehydroxylation of the metal hydroxide layers. Furthermore, at 723 K, split peaks are observed at 1534 and 1385 cm^–1^, which are likely assigned to CO_3_^2–^ coordinating to metals.

To summarize the behavior of Co–Al LDH, H_2_O and CO_2_ are released in three distinct steps (Figure 9a). Based on the discussion in the previous section, it can be concluded that the chemical reactions in Co–Al LDH are analogous to those in Mg–Al LDH and Zn–Al LDH except the changes in the coordination state of CO_3_^2–^. These reactions involve the release of interlayer H_2_O molecules (Step (1)), partial dehydroxylation of the metal hydroxide layers, and complete dehydroxylation of the layers along with the decomposition of interlayer CO_3_^2–^ (Step (3)). For the second step of Co–Al LDH, we could not detect the coordination of CO_3_^2–^ to metal ions by the in situ FT-IR measurements (Figure 10b). That is the difference from Step (2) in Mg–Al LDH and Zn–Al LDH. Moreover, the in situ XRD results (Figure 10a) suggest that Co–Al LDH lost the layered structure in Step (1). This structural transformation sets it apart from Mg–Al LDH, which finally loses its layered structure in Step (3). In addition, the decomposition behavior is affected by the atmosphere, shifting from three steps to two steps depending on the presence of oxygen, likely due to the oxidation of Co^2+^.

### Comparison of Preliminary First-Principles DFT Calculations with Experiments

In this discussion, we will compare the preliminary calculations (Figures 2 and 3) with the experimental results. Here we should note that the structural models (single layer slab models in Figures 2 and 3) are much simplified and the energy values obtained in the calculations are not suitable for direct comparison to experimental thermodynamic data. The nature of the divalent cations (Mg, Zn, and Co) may be reflected in the relative calculated values and/or the order of the values. Let us begin by examining the dehydroxylation reaction. The calculated reaction energies follow this order: Co–Al LDH (225 kJ mol^–1^) < Zn–Al LDH (266 kJ mol^–1^) < Mg–Al LDH (344 kJ mol^–1^). Figures 6a, 7a and 9a show that the initial temperature of dehydroxylation reaction for Mg–Al LDH was 552 K, for Zn–Al LDH, it was 497 K, and for Co–Al LDH, it was 520 K. The inconsistency between the calculated and experimental results can be attributed to the assumption made during the calculations. The calculations assumed the preservation of the layered structure during the dehydroxylation reaction, even though Zn–Al LDH and Co–Al LDH lose their layered structure before the dehydroxylation. This difference in conditions may have affected the calculation results. Moving on to the decomposition reaction of CO_3_^2–^ coordinating with metal hydroxide layers, we observe the following order in calculated reaction energies: Co–Al LDH (86 kJ mol^–1^) < Zn–Al LDH (143 kJ mol^–1^) < Mg–Al LDH (171 kJ mol^–1^). These calculation results partially consistent with the experimental results. Specifically, Mg–Al LDH dehydroxylates and releases CO_2_ at the highest temperature among the three LDH samples, consistent with our expectations based on calculations. However, there is a discrepancy in the order of reaction temperatures between the calculation and experiment for Zn–Al LDH and Co–Al LDH. Interestingly, the experimental results align with the order of decomposition enthalpies of carbonates of divalent metal ions in each LDH.^46^ The decomposition temperatures of carbonate species follow the Fajans rules for metal ions. Considering that the layered structure decomposed in Step (1) for Zn–Al LDH and Co–Al LDH, the chemical environment of the CO_3_^2–^ ions is likely to be much different from that in the interlayer spaces of LDHs. For Zn–Al LDH and Co–Al LDH the decomposition temperatures of interlayer CO_3_^2–^ in LDHs are determined by the polarizing ability of the metal ions constituting the LDHs, with higher polarizing ability resulting in lower decomposition temperatures. The inconsistency between calculated and experimental results likely stems from the assumption of preserving the layered structure during CO_3_^2–^ decomposition in the calculations. Of course, reaction rates are governed by the activation energies and not by the reaction energies. Thus, elucidation of the transition states and estimation of the activation energies is an important subject for future theoretical study.

### Quantitative Aspects of Multi-Step Chemical Transformation of LDH Samples at Elevated Temperatures

Furthermore, we have determined the amounts of H_2_O and CO_2_ evolutions separately in each step of the multistep transformation for three LDH samples. First, we quantified the amounts of H_2_O and CO_2_ evolutions of Mg–Al LDH, and the results are listed in Table 2. These values were determined based on the quantification of CO_2_ gas evolution in Figure 6a and the weight losses in Figure 6b. According to Table 2, the total amount of evolved CO_2_ is 1.640 mmol g^–1^ over the entire temperature range. However, total amount of the initial interlayer CO_3_^2–^ in the LDH sample was calculated to be 2.131 mmol g^–1^, suggesting the possibility that a small amount of CO_3_^2–^ remains after the decomposition of the LDH at 1073 K. Assuming this remaining amount of CO_3_^2–^, the chemical composition of the sample after decomposition at 1073 K is described as Mg_0.671_Al_0.329_O_1.1264_(CO_3_)_0.0379_.

**Table 2:** Amounts of H_2_O and CO_2_ Released During Multi-Step Transformation of Mg–Al LDH

quantified values	first step	second step	third step and higher temp.
weight loss/%	12.8	7.1	21.1
CO_2_ evolution/mmol g^–1^	0.026	0.040	1.586
CO_2_ evolution/molecule metal ion^–1^	0.002	0.002	0.122
H_2_O evolution/mmol g^–1^	7.040	3.856	7.857
H_2_O evolution/molecule metal ion^–1^	0.543	0.297	0.606

From the final chemical composition and the evolution amounts of H_2_O and CO_2_ in each step summarized in Table 2, we have determined the corresponding chemical compositions of the sample, shown in Scheme S1 in the Supporting Information. Notably, the chemical composition before the second step is Mg_0.671_Al_0.329_O_0.0991_(OH)_1.8060_(CO_3_)_0.1622_ (Scheme S1), indicating that all interlayer H_2_O molecules have been released. The small amount of oxide ions arises due to dehydroxylation of the metal hydroxide layers and the decomposition of interlayer CO_3_^2–^. These results indicate that in the first step, the majority of the weight loss is mostly due to the release of interlayer H_2_O molecules and the dehydroxylation reaction is minor. This understanding of the structural/chemical transformation of Mg–Al LDH provides valuable insights for discussing the behaviors of Co–Al LDH and Zn–Al LDH at elevated temperatures in the following sections.

Moving on, we have determined the amounts of H_2_O and CO_2_ evolution separately in each step of the multistep transformation for Zn–Al LDH, similar to the approach applied to Mg–Al LDH, as described in the previous section. The values in Table 3 were determined based on Figures 7a and 7b. The chemical composition of the sample after decomposition at 1073 K was determined to be Zn_0.66_Al_0.34_O_1.108_(CO_3_)_0.059_.

**Table 3:** Amounts of H_2_O and CO_2_ Released During Multi-step Transformation of Zn–Al LDH

		first step	second step	third step	higher temp.
quantified values	rt–397 K	397–480 K	480–512 K	512–763 K	763–1073 K
weight loss/%	7.6	13.9	3.6	12.4	3.4
CO_2_ evolution/mmol g^–1^	0.007	0.022	0.040	0.47	0.34
CO_2_ evolution/molecule metal ion^–1^	0.001	0.003	0.005	0.059	0.042
H_2_O evolution/mmol g^–1^	4.196	7.639	1.890	5.765	1.042
H_2_O evolution/molecule metal ion^–1^	0.523	0.952	0.236	0.719	0.130

Using the values listed in Table 3, we have calculated the chemical composition of the sample for each chemical transformation step, as shown in Scheme S2. The chemical composition before the second step is Zn_0.663_Al_0.337_O_0.0036_(OH)_2_(CO_3_)_0.1648_·0.0843H_2_O (Scheme S2). In this composition, the generation of oxide ions is ascribed to the decomposition of a small amount of interlayer CO_3_^2–^. This composition indicates that the H_2_O evolution in the first step is solely ascribed to the release of interlayer H_2_O molecules, with the majority (94.6%) of interlayer molecules being released in this step. The results indicate no evidence of the dehydroxylation reaction of the hydroxide layers in the first step, so the number of hydroxyl groups in the chemical composition is just 2 (twice the total number of metal ions). This analysis clearly indicates that Zn–Al LDH undergoes the same three-step chemical transformations as Mg–Al LDH: release of interlayer H_2_O molecules (Step (1)), partial dehydroxylation of the metal hydroxide layers (Step (2)), and complete dehydroxylation of the layers and decomposition of interlayer CO_3_^2–^ (Step (3)).

Until now, there have been no studies that have quantitatively defined both H_2_O and CO_2_ evolution separately. Our results allow for quantitative discussion, revealing that the layered structure is lost while the hydroxyl groups remain. In addition, we have clarified that the chemical transformation behaviors for Zn–Al LDH are essentially the same as Steps (1–3) for Mg–Al LDH, although there is a structural transformation difference involving the loss of the layered structure due to the release of interlayer H_2_O molecules.

In a manner similar to the previous sections for Mg–Al LDH and Zn–Al LDH, we have determined the amounts of H_2_O and CO_2_ evolutions separately for each step of transformation of Co–Al LDH. The values presented in Table 4 were determined based on the quantification of CO_2_ gas evolution in Figure 9a and the weight losses in Figure 9b. The chemical composition of the sample after decomposition at 1073 K is calculated to be Co_0.639_Al_0.361_O_1.0989_(CO_3_)_0.0816._.

**Table 4:** Amounts of H_2_O and CO_2_ Released During Multi-step Transformation of Co–Al LDH

	first step	second step	third step	higher temp.
quantified values	rt–488 K	488–559 K	559–684 K	684–1073 K
weight loss/%	10.8	5.1	11.8	3.3
CO_2_ evolution/mmol g^–1^	0.053	0.013	0.62	0.29
CO_2_ evolution/molecule metal ion^–1^	0.005	0.001	0.064	0.030
H_2_O evolution/mmol g^–1^	5.867	2.779	5.140	1.168
H_2_O evolution/molecule metal ion^–1^	0.607	0.287	0.531	0.121

Using the values listed in Table 4, we have calculated the chemical composition of the sample for each chemical transformation step, as shown in Scheme S3. The chemical composition before the second step is Co_0.639_Al_0.361_O_0.0977_(OH)_1.8153_(CO_3_)_0.1751_ (Scheme S3). This result indicates that all of the interlayer H_2_O molecules are released. The small amount of oxide ion is due to the dehydroxylation of the metal hydroxide layers and the decomposition of interlayer CO_3_^2–^. In the first step, most of the weight loss is due to the release of interlayer H_2_O molecules, similar to Mg–Al LDH. This analysis indicates that Co–Al LDH undergoes the same three-step chemical transformations as Mg–Al LDH and Zn–Al LDH: release of interlayer H_2_O molecules, partial dehydroxylation of the metal hydroxide layers, and complete dehydroxylation of the layers and decomposition of interlayer CO_3_^2–^. Therefore, the first and third steps for Co–Al LDH correspond to Step (1) and (3) for Mg–Al LDH, respectively. For the second step, the coordination state of CO_3_^2–^ is unclear in Co–Al LDH, and this is the difference from Step (2) in Mg–Al LDH and Zn–Al LDH.

## Conclusions

In this study, we have conducted a detailed analysis of the thermal decomposition behaviors of *M*–Al LDH samples, where *M* = Mg, Co, and Zn. We have leveraged quantitative analysis techniques which involve determining the evolution amounts of H_2_O and CO_2_ in each reaction step at elevated temperatures by quantifying CO_2_ gas evolution and measuring TGA. Our findings reveal that despite variations in the reaction temperatures for each step, the chemical transformations for Zn–Al LDH, Co–Al LDH, and Mg–Al LDH occurred in the three steps: the release of interlayer water (Step (1)), partial dehydroxylation of metal hydroxide layers, and complete dehydroxylation of layers and decomposition of interlayer CO_3_^2–^ (Step (3)). In the cases of Mg–Al LDH and Zn–Al LDH, the second step involve coordination of CO_3_^2–^ to metal ions (i.e., Step (2)). Furthermore, it is important to note that the structural transformations for each sample differ significantly: the layered structure collapses in Steps (1) for Zn–Al LDH and Co–Al LDH, and Step (3) for Mg–Al LDH. Our ability clearly elucidate the decomposition behavior for Mg–Al LDH, along with the quantitative analysis of CO_2_ evolution that has hardly been applied to LDHs by researchers and in situ measurements, has enabled us to obtain an interpretation for the chemical and structural transformations in LDHs. This study sheds light on how the nature of divalent metal ions in LDHs influences their thermal decomposition. This fundamental information on the chemical nature of these materials are essential to explore functional applications such as CO_2_ adsorbents. Notable subjects for future studies include the design and control of gas desorption behaviors from LDH-based adsorbents based on the atomic/molecular-level interpretation of the chemical transformation of these materials at elevated temperatures.

## Supporting Information Available

It includes magnified XRD patterns, Surface textural images by FE-SEM, Raman spectra, peak separation results for in situ FT-IR spectra for Zn–Al LDH, and reaction schemes for Mg–Al LDH, Zn–Al LDH, and Co–Al LDH (PDF)
