# Supplementary material for: Chemical and Structural Transformations of M–Al–CO3 Layered Double Hydroxides (M = Mg, Zn, or Co, M/Al = 2) at Elevated Temperatures: Quantitative Descriptions and Effect of Divalent Cations
Source: Inorg Chem. 2024 Aug 12;63(34):15634–47. doi: 10.1021/acs.inorgchem.4c01186 (PMC11351178; doi:10.1021/acs.inorgchem.4c01186)
Supplement: Supplementary file 1 — ic4c01186_si_001.pdf [file ic4c01186_si_001.pdf]

Supporting Information.

Chemical and Structural Transformations of *M*–Al–CO_3 Layered Double Hydroxides (*M* = Mg, Zn, or Co, *M*/Al = 2) at Elevated Temperatures: Quantitative Descriptions and Effect of Divalent Cations

Kaito Matsuda [a], Ayaka Okuda [a], Nana Iio [a], Naoki Tarutani [a]*, Kiyofumi Katagiri [a], and Kei Inumaru [a]*

[a] *Graduate School of Advanced Science and Engineering, Hiroshima University, 1-4-1, Kagamiyama, Higashihiroshima, Hiroshima 739-8527, Japan.*

Corresponding Authors:
Naoki TARUTANI; n-tarutani@hiroshima-u.ac.jp
Kei INUMARU; inumaru@hiroshima-u.ac.jp

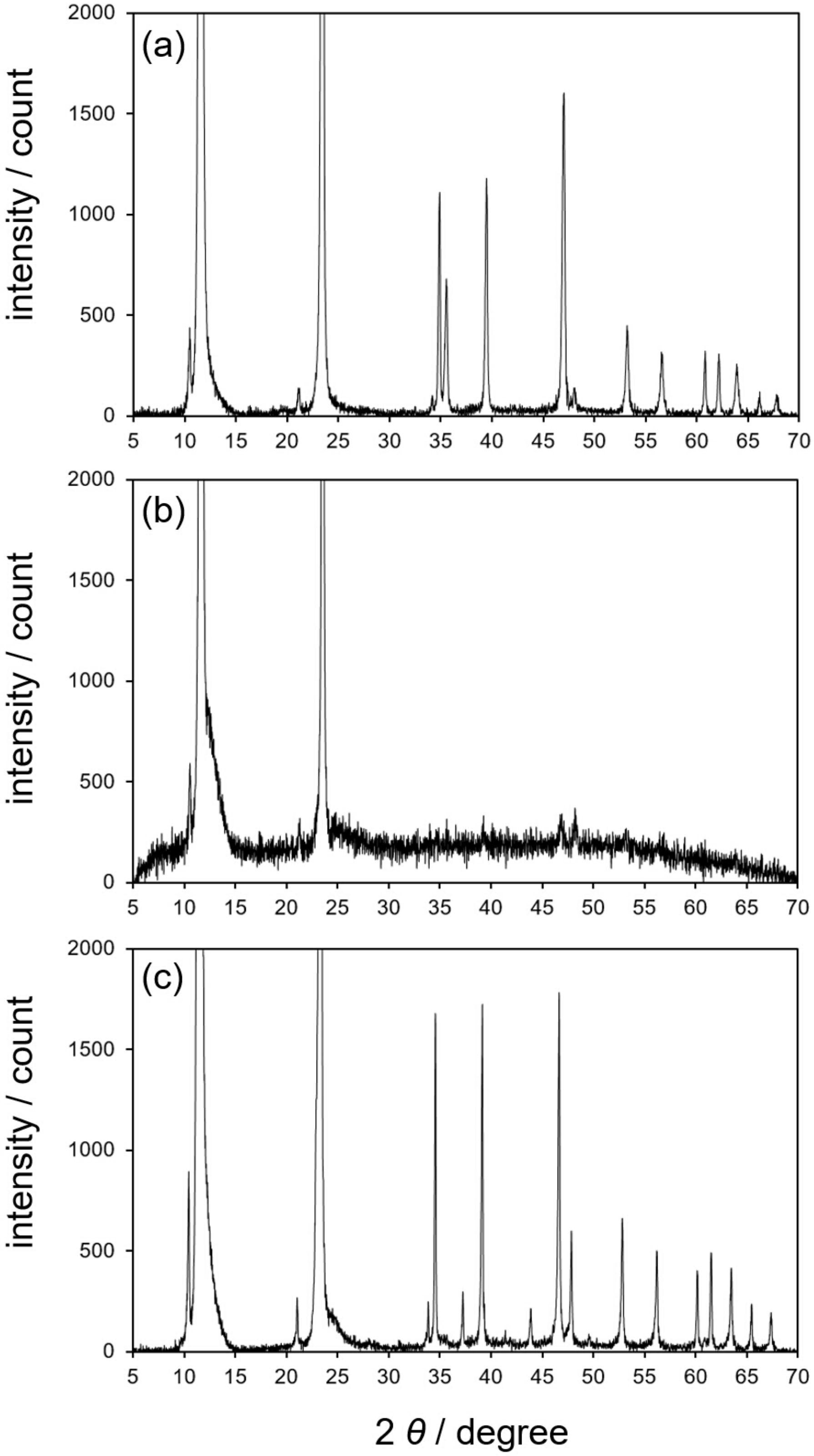

Figure S1. Magnified XRD patterns of LDH samples. (a) Mg–Al LDH. (b) Co–Al LDH. (c) Zn–Al LDH.

Comparison of (b) Co–Al LDH with Figure 10a in the main article indicates that the data in (b) is affected by preferred orientation of the sample particles in this measurement.

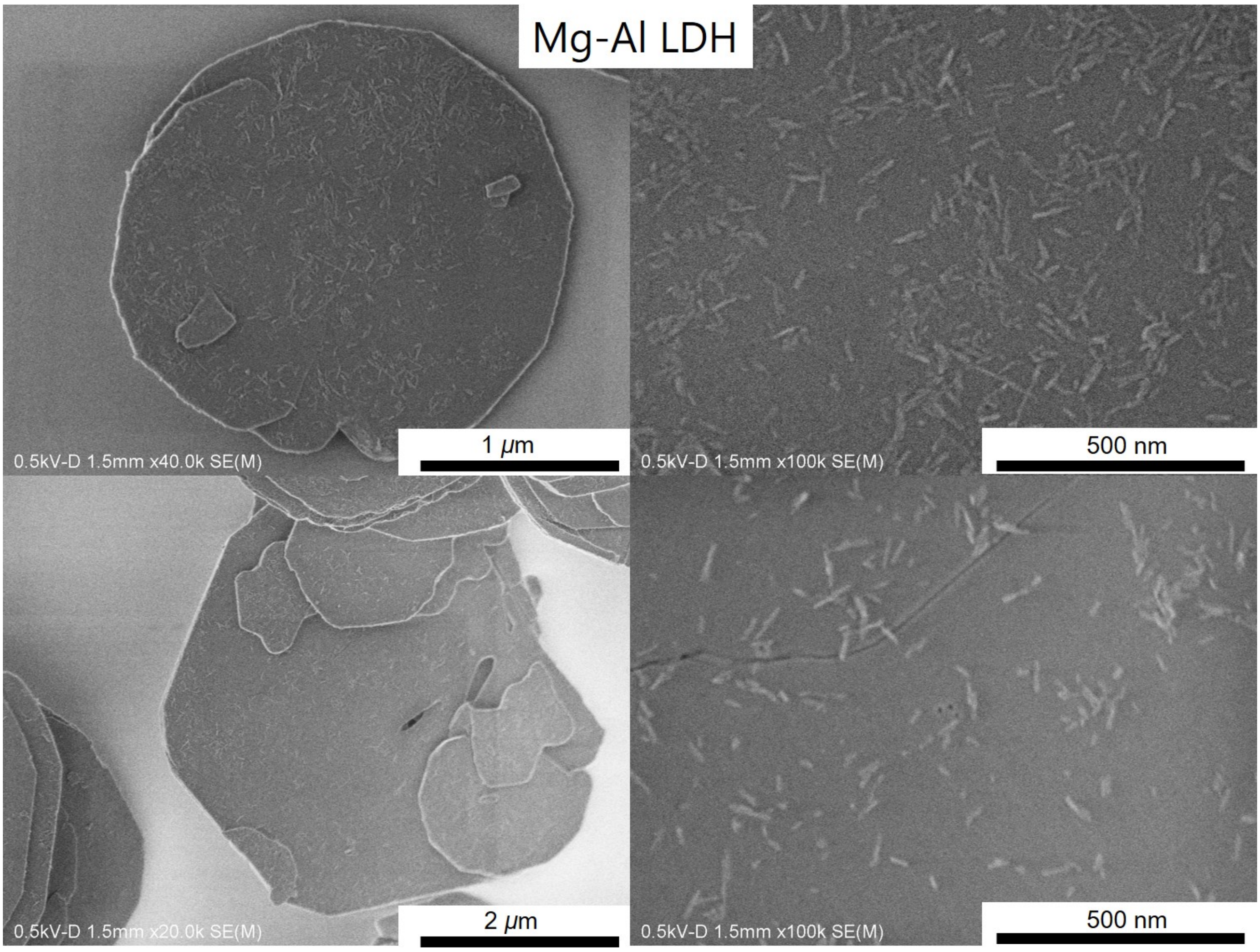

Figure S2. Surface textural FE-SEM images of Mg–Al LDH taken at a low acceleration voltage.

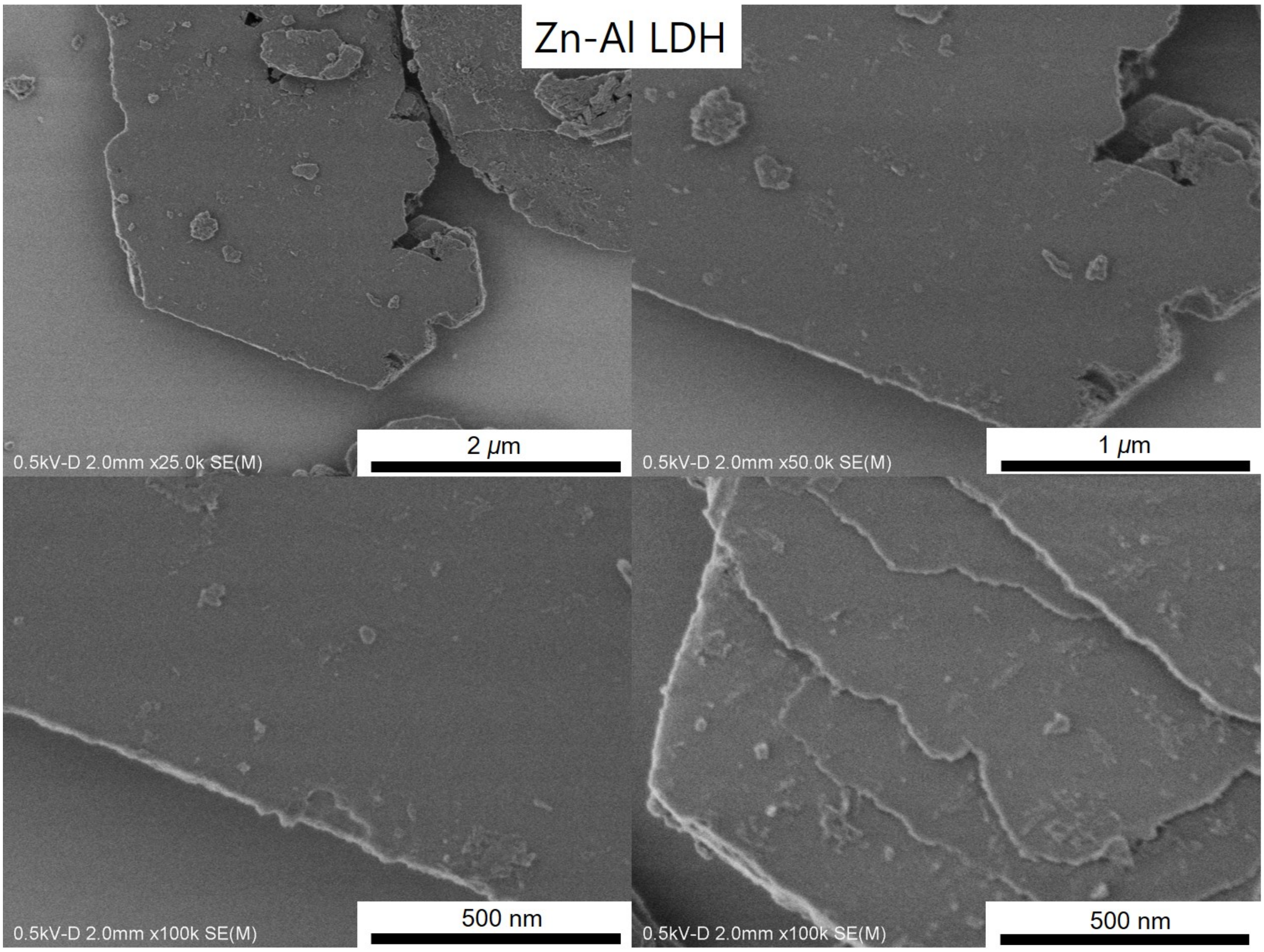

Figure S3. Surface textural FE-SEM images of Zn–Al LDH taken at a low acceleration voltage.

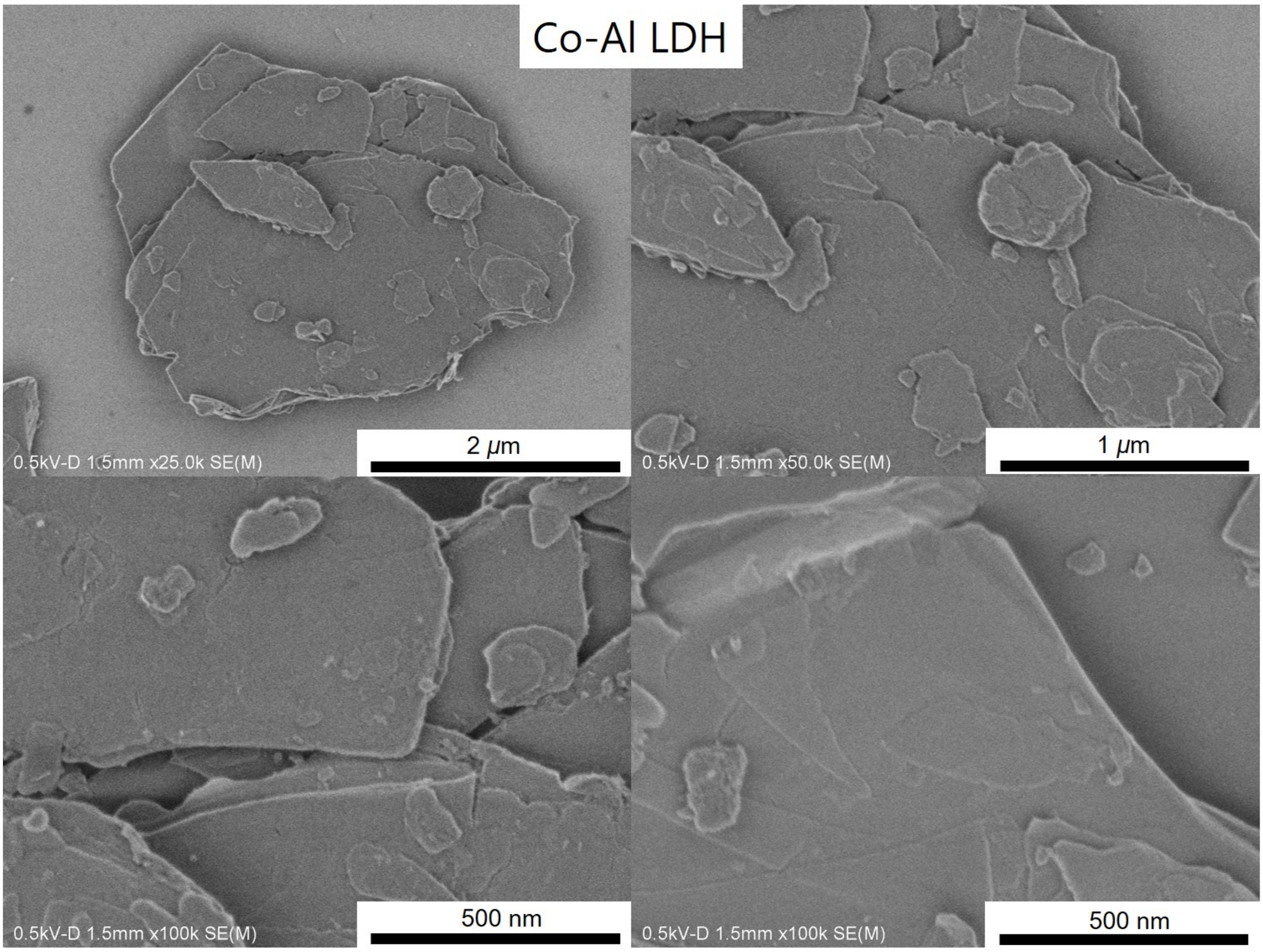

Figure S4. Surface textural FE-SEM images of Co–Al LDH taken at a low acceleration voltage.

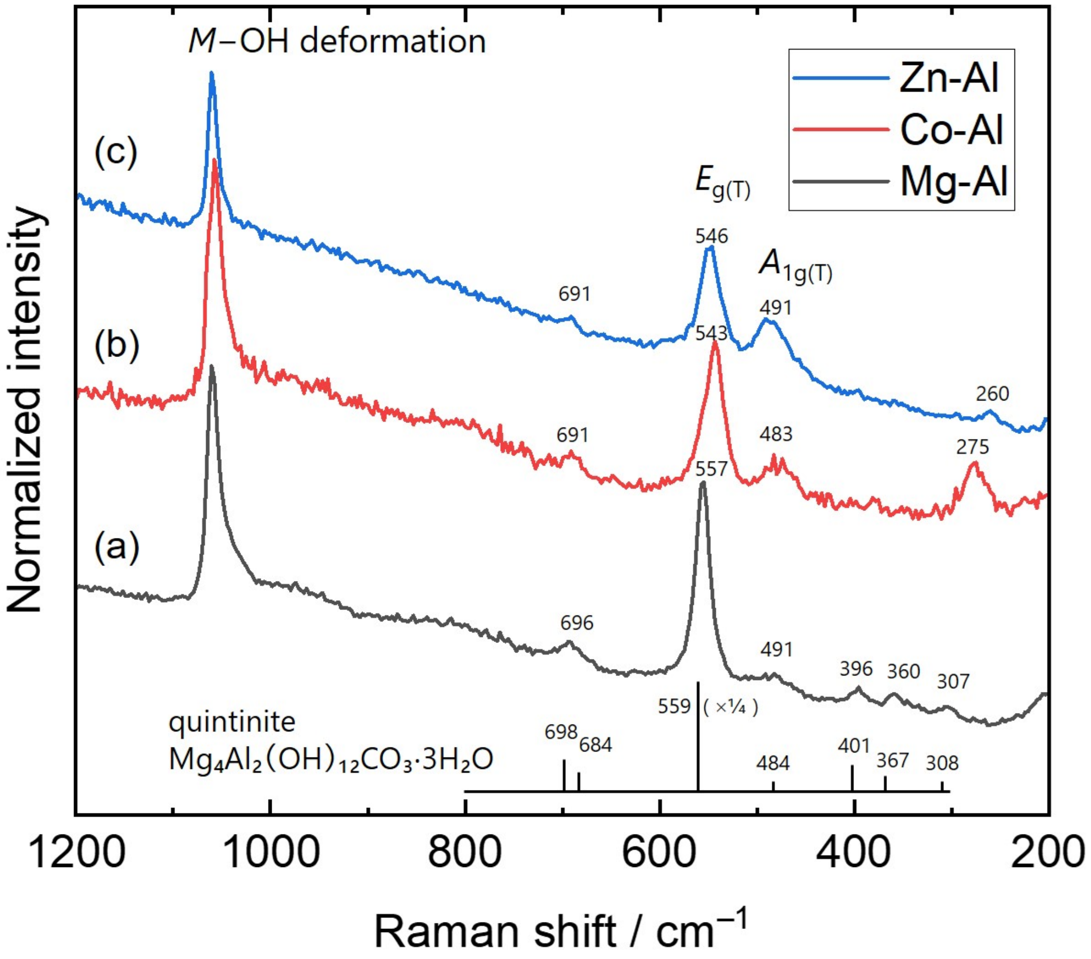

Figure S5. Raman spectra of LDH samples. (a) Mg–Al LDH. (b) Co–Al LDH. (c) Zn–Al LDH. The bar chart represents spectral data of well-crystallized quintinite $Mg_4Al_2(OH)_{12}CO_3 \cdot 3H_2O$ [ref].

[Ref.] Theiss, F.; López A.; Frost, R. L.; Scholz, R. Spectroscopic Characterisation of the LDH Mineral Quintinite $Mg_4Al_2(OH)_{12}CO_3 \cdot 3H_2O$, *Spectrochimica Acta A*, **2015**, *150*, 758−764.

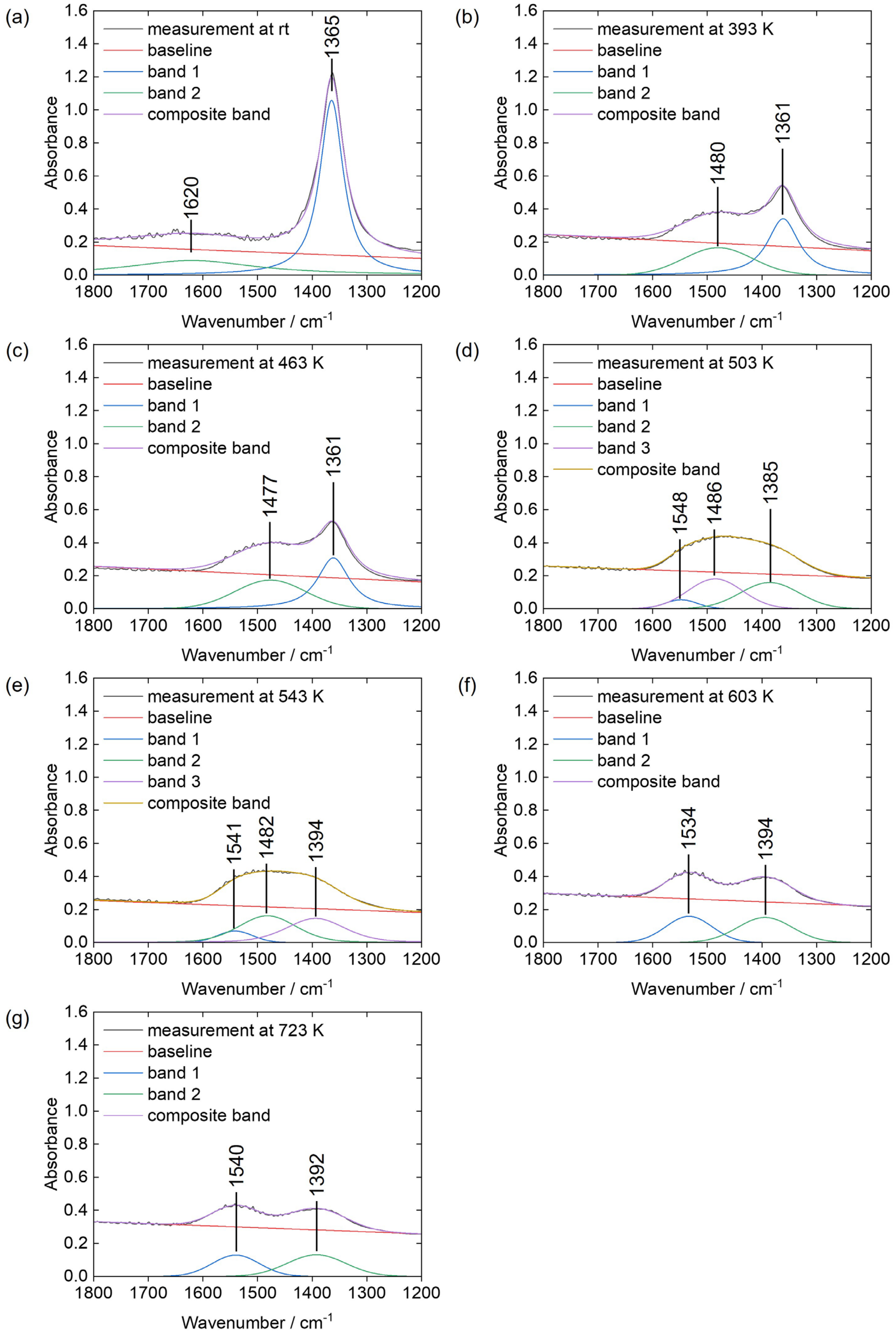

Figure S6. Peak fitting results of *in situ* FT-IR spectra for Zn–Al LDH shown in Figure 7b in vacuum at elevated temperatures. (a) room temperature, (b) 393 K, (c) 463 K, (d) 503 K, (e) 543 K, (f) 603 K and (g) 723 K.

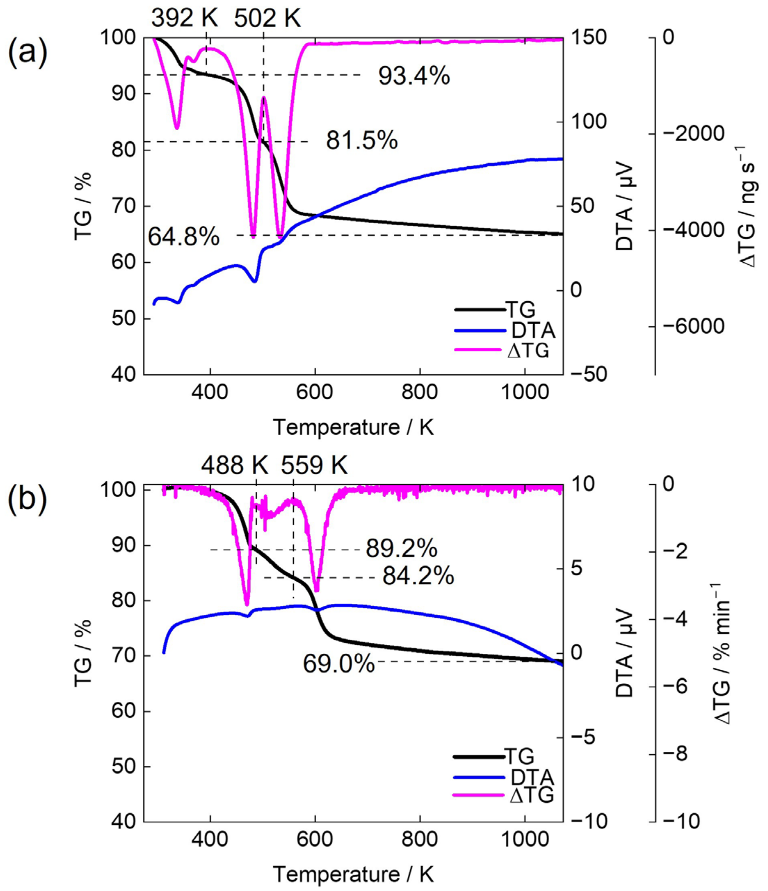

Figure S7. TG-DTA-DTG curves for Co–Al LDH in (a) an air flow and (b) a He flow.

Scheme S1. Change of composition and weight loss of Mg–Al LDH in each step.

$Mg_{0.671}Al_{0.329}(OH)_2(CO_3)_{0.1643} \cdot 0.45H_2O$

1st step ↓ (−12.8%,rt–513 K)

$Mg_{0.671}Al_{0.329}O_{0.0991}(OH)_{1.8060}(CO_3)_{0.1622}$

2nd step ↓ (−7.1%, 513–630 K)

$Mg_{0.671}Al_{0.329}O_{0.3984}(OH)_{1.2115}(CO_3)_{0.1602}$

3rd step ↓ (−21.1%, 630 K–)

$Mg_{0.671}Al_{0.329}O_{1.1264}(CO_3)_{0.0379}$

Scheme S2. Change of composition and weight loss of Zn–Al LDH in each step.

$Zn_{0.663}Al_{0.337}(OH)_2(CO_3)_{0.1684}\cdot 1.5600H_2O$

Desorption of surface H_2O↓ (−7.6%, rt–397 K)

$Zn_{0.663}Al_{0.337}O_{0.0008}(OH)_2(CO_3)_{0.1675}\cdot 1.0368H_2O$

1st step ↓ (−13.9%,397–480 K)

$Zn_{0.663}Al_{0.337}O_{0.0036}(OH)_2(CO_3)_{0.1648}\cdot 0.0843H_2O$

2nd step ↓ (−3.6%, 480–512 K)

$Zn_{0.663}Al_{0.337}O_{0.1596}(OH)_{1.6980}(CO_3)_{0.1598}$

3rd step ↓ (−12.5%, 512–763 K)

$Zn_{0.663}Al_{0.337}O_{0.9376}(OH)_{0.2601}(CO_3)_{0.1007}$

Higher temp. ↓ (−3.4%, 763 K–)

$Zn_{0.663}Al_{0.337}O_{1.1099}(CO_3)_{0.0585}$

Scheme S3. Change of composition and weight loss of Co–Al LDH in each step.

$Co_{0.639}Al_{0.361}(OH)_2(CO_3)_{0.1805} \cdot 0.50H_2O$

1st step ↓ (−10.8%,rt–488 K)

$Co_{0.639}Al_{0.361}O_{0.0977}(OH)_{1.8153}(CO_3)_{0.1751}$

2nd step ↓ (−5.1%, 488–684 K)

$Co_{0.639}Al_{0.361}O_{0.3803}(OH)_{1.2527}(CO_3)_{0.1739}$

3rd step ↓ (−11.8%, 559–684 K)

$Co_{0.639}Al_{0.361}O_{0.9552}(OH)_{0.2287}(CO_3)_{0.1110}$

Higher temp. ↓ (−3.3%, 684 K–)

$Co_{0.639}Al_{0.361}O_{1.0989}(CO_3)_{0.0816}$
